# SQSTM1/p62 promotes miR-198 loading into extracellular vesicles and its autophagy-related secretion

**DOI:** 10.1007/s13577-022-00765-7

**Published:** 2022-09-01

**Authors:** Xiaojie Yu, Hannah Eischeid-Scholz, Lydia Meder, Vangelis Kondylis, Reinhard Büttner, Margarete Odenthal

**Affiliations:** 1 Faculty of Medicine, Institute for Pathology and University Hospital Cologne, University of Cologne, 50924 Cologne, Germany; 2 Faculty of Medicine, Center for Molecular Medicine Cologne (CMMC), and University Hospital Cologne, University of Cologne, 50924 Cologne, Germany; 3 Faculty of Medicine Department I of Internal Medicine, University Hospital Cologne, University of Cologne, 50931 Cologne, Germany; 4 Cologne Excellence Cluster On Cellular Stress Responses in Aging-Associated Diseases (CECAD), University of Cologne, 50931 Cologne, Germany; 5 Faculty of Medicine, Center of Integrative Oncology and University Hospital Cologne, University of Cologne, 50924 Cologne, Germany

**Keywords:** EV, HCC, microRNA, SQSTM1, Autophagy

## Abstract

MicroRNA dysregulation is a hallmark of hepatocellular carcinoma (HCC), leading to tumor growth and metastasis. Previous screening on patient specimens identified miR-198 as the most downregulated miRNA in HCC. Here, we show that miR-198 compensation leads to self-release into extracellular vesicles (EVs). Importantly, the vesicular secretion is mediated by autophagy-related pathway, initiated by sequestration of p62/miR-198 complexes in autophagosome-associated vesicle fractions. miR-198 is selectively recognized and loaded by p62 into autophagosomal fractions, whereas mutated miR-198 forms neither induce autophagy and nor interact with p62. Gain and loss of function experiments, using a CRIPR/Cas knockout (KO) and transgenic site-specific p62 mutants, identified p62 as an essential repressor of cellular miR-198 abundancy. Notably, EVs, harboring miR-198/p62 protein complexes, can be uptaken by cells in the close vicinity, leading to change of gene expression in recipient cells. In conclusion, miR-198 enhances autophagy; conversely autophagic protein p62 reduces the miR-198 levels by sorting into extracellular space.

**Graphical abstract:**

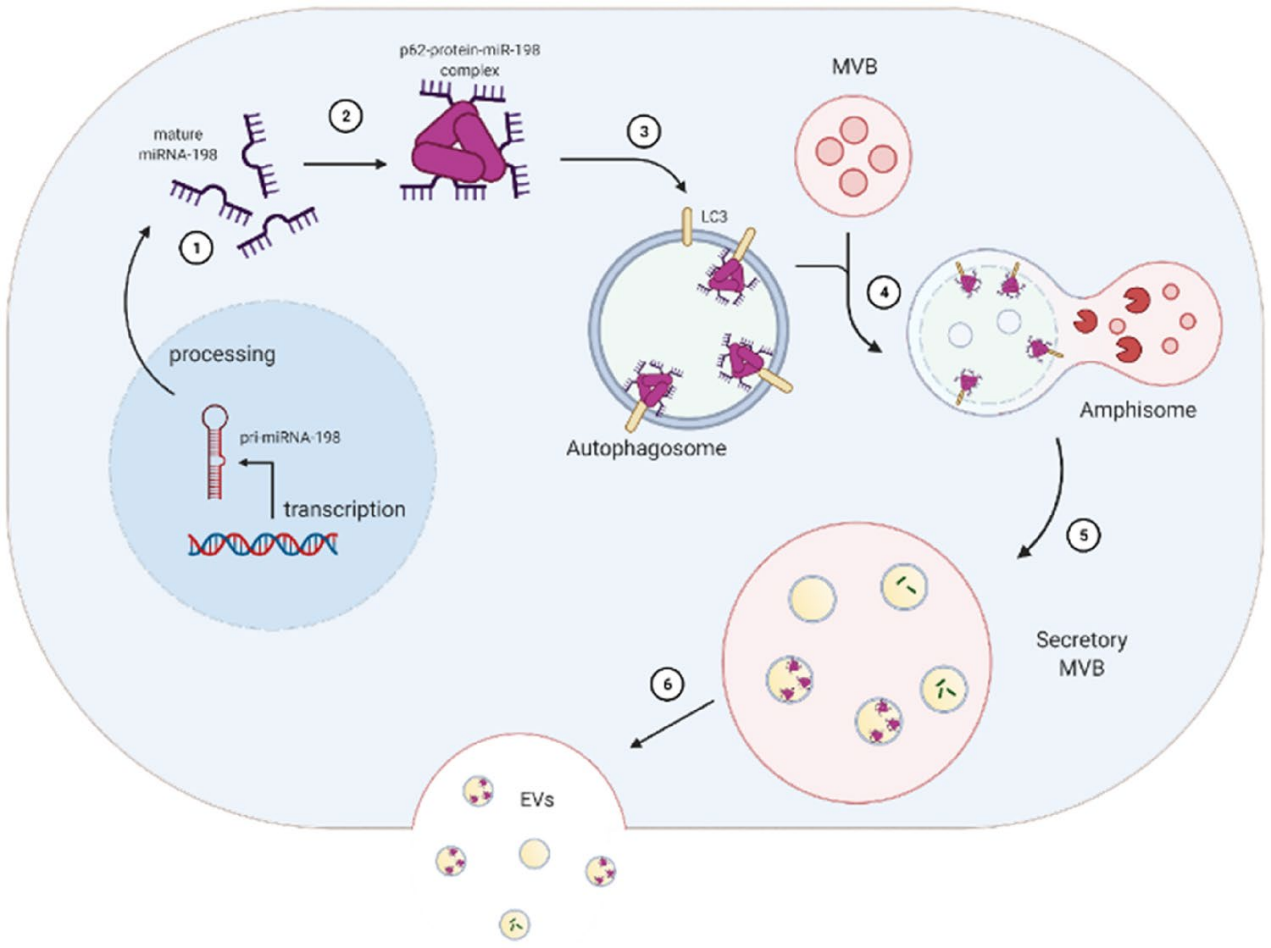

miR-198 is at first transcribed as primary miRNA, after being processed into single stranded mature miR-198 form, it is transported into cytoplasm ①. By interaction with p62 protein, miR-198 conglomerates and forms a binding complex ②. Since LC3 protein is an interaction partner of p62 protein, hence miR-198 is included into autophagosomes ③. By fusion with multivesicular bodies (MVB), miR-198-binding complex was recruited into amphisomes ④, the latter of which quickly turns into secretory MVB containing intraluminal vesicles⑤. By fusion with cell membrane, intraluminal vesicles were released into extracellular space as EVs ⑥.

**Supplementary Information:**

The online version contains supplementary material available at 10.1007/s13577-022-00765-7.

## Introduction

Although macroautophagy (hereafter referred as autophagy) is classically considered as a degradative process [1], accumulative evidences have implicated its role in secretion of pro-inflammatory cytokines [2], lysozyme release into extracellular environment [3], and vesicle production [4]. These processes [2–4] are collectively termed as autophagy-associated vesicle secretion, indicating an autophagy pathway steadily maintaining cell homeostasis and shaping tissue microenvironment under both normal and pathological conditions. The concept of secretory autophagy is recently described and is not well understood until now. Most of the studies focus on the recruitment of cargos into autophagosomes, but a limited number of them investigate autophagy-associated vesicle release [2, 5, 6]. Moreover, the evidences were mostly presented as the effect of changes of autophagic activity, either by inhibition or by induction, on protein secretion [2, 5, 6], however, none of the molecular mechanisms regarding the cargo loading, vesicle release and uptake were revealed until now.

As autophagy scaffold protein, SQSTM1/p62 protein loads cargos into autophagosomes by interacting with LC3 protein [7]. Under physiological conditions, p62 protein was barely detectable due to rapid degradation in lysosomal compartment during autophagy process [8]. However, in hepatocellular carcinoma (HCC), p62 protein was highly enriched implicating the impairment of degradative autophagy [9], the latter of which was shown to trigger miRNA dysregulation in Hungtington disease [10]. In parallel, aberrant miRNA expression is one of the major characteristics in HCC where tumor suppressor miRNAs are generally downregulated and oncogenic ones are upregulated [11].

MicroRNAs (miRNAs) are small, endogenous oligonucleotides, typically 18–24nt long. They bind to the 3'-untranslated region (3'-UTR) of transcripts and repress the expression of target genes. Among the screened 80 miRNAs, the primate specific miR-198 is the most downregulated in HCV-associated liver cancer and classified as one potent tumor suppressor [12, 13]. To note, the expression of miR-198 was gradually decreased with the progress of HCV-related liver diseases (including fibrosis, cirrhosis, dysplastic nodule and HCC) [12] and it was nearly undetectable in hepatoma cells in contrast to highly enrichment in parenchymal liver tissues [13]. In parallel, miR-198 was released into blood serum of HCV-infected patients at different stages [14] and it was also detected in the serum of patients with HCC or liver cirrhosis [15], pointing to the link between cellular decrease and its release into extracellular space. Importantly, EV is the major vehicle for miRNA carrier for the release [16–18], however, few of them have studied the mechanism of packaging miRNA into vesicles.

Here, we describe the autophagy-associated vesicle release mechanism, by which p62 selectively recruits miR-198 into autophagosome-derived vesicular fraction and secretes into EVs. The vesicular miR-198 is uptaken up by recipient cells, in which it is functional, leading to alteration of targeted miR-198 sensor reporter expression. Furthermore, we demonstrate that p62 is a strong repressor for miR-198 accumulation, presenting as inversely regulating miR-198 levels. Importantly, we show that p62 or autophagy massively antagonizes the suppressive function of HCC cell growth by miR-198. Thus, p62 dependent autophagy-mediated secretion emerges as an essential mechanism for miR-198 dysregulation.

## Materials and methods

### Cell lines

HCC cells (HuH-7, Hep3B, kindly provided by Ulrike Protzer in 2008)) were cultured in DMEM supplemented with 10% heat inactivated FCS (PAN, #P30-8500). We established HuH-7/ Hep3B Tet-On-miR-198 cell line using the Mir-X™ Inducible miRNA System (Takara, #631,120), and prepared stably expressing doxycycline-inducible cell lines following manufacturer’s instructions. Stable polyclonal cells were grown in DMEM medium containing 10% Tet-On approved FBS (Takara, #631,106). Furthermore, we used the Hepa1-6 (ATCC, mouse origin), HSC-T6 (rat origin) and LX-2 cells (human origin), both kindly provided by Scott Friedman since 2009, were maintained in DMEM as above. All the cell lines have been recently authenticated with deep sequencing. All experiments were performed with exosome-free FCS (Thermo Fischer, #A2720801) unless indicated otherwise.

### Patient biopsies

All FFPE biopsy specimens were collected by the Institute of Pathology at University Hospital of Cologne (Cologne, Germany) were utilized in accordance with the policy of institutional review board of the hospital (18–052). The histology and immunohistology were made under blinded basis and confirmed by pathologists as described earlier [12].

### Generation of miR-198 overexpression systems

For Tet-On inducible expression system: HuH-7 and Hep3B cells were transfected by Jetprime transfection reagent (Polyplus, #114–15) with two plasmids, harboring the expression of Tet-On inducible expression system (Takara, #631,120). Pri-miR-198 encoding sequence was cloned in the downstream of Tet-on promoter. The two plasmids were fused into one vector by molecular cloning. Cells were transfected with the fused plasmid coding Tet-On miR-198 expression cassette and then selected by G418 for 14 d. Monoclones were isolated by serial dilution in 96-well plate. Here, mock plasmid without miR-198 expression was used as control. For induction, stably transfected cells were treated by dox at final concentration of 1 ng/ml.

For pCMV stable expression system: cells were transfected using the plasmid carrying miR-198 expression cassette under CMV promoter and selected by G418 for 14 d. Here, polyclonal cells were used for further experiment.

### Generation of p62 knockout cell line by the Crispr/Cas technology

For the generation of p62 knockout cell line, single guide RNAs targeting the p62 locus were predicted using the CRISPR online tool (http://crispor.tefor.net; Version 4.98) [19], ordered from Sigma-Aldrich, annealed, and cloned into pSpCas9(BB)-2A-puro (PX459) (Addgene, #62,988). The generated plasmids were transfected into HuH-7 cells using the Jetprime transfection reagent (Polyplus, #114–15) according to manufacturer’s instructions. As negative control parental plasmid (without sgRNA) was used. Cells were treated with puromycin for 14 d and single clones were isolated by serial dilution in 96-well plate.

### RNA isolation

The isolation of total RNA from mammalian cells was performed using the TRIZOL method. Trizol (Sigma, #T9424) was used to lyse the cells and chlorophorm isoamylalcohol was added and incubated for centrifugation. The aqueous phase was transferred to a fresh tube and precipitated using isopropanol. The RNA pellet was washed with ethanol, air-dried and resuspended in RNase-free water. RNA concentration was determined by A_260_-measurement using the spectrophotometer (NanoDrop, #ND-1000) and quality was measured by microcapillary electrophoresis (2100 BioAnalyser, Agilent Technologies).

For RNA isolation from supernatant or EVs, prior isolation, 1 pmol/100 µl spike-in RNA was added directly in the sample and RNA was isolated using TRIZOL method.

### Quantitative real-time PCR (qPCR)

MicroRNA was analyzed by a two-step qPCR using the miScript-Reverse Transcription Kit (Qiagen, #218,161) and GoTaq®qPCR Master Mix (Promega, #A6001) and primer sets. Primers used for cDNA synthesis and real-time PCR were selected and purchased from the GeneGlobe Search Center (Qiagen). All steps were performed in triplicate and in agreement with the supplier’s guidelines. After demonstration that primer sets exert equal and high efficiencies, relative expression was calculated by the ∆∆Ct method using the transcript levels of hypoxanthine–guanine phosphoribosyl transferase (HPRT) for normalization. Cellular miRNA levels were normalized using RNU6 as reference. For normalization of extracellular miRNA levels, SV40-miRNA and *C.elegans* miRNA (Qiagen) were added to samples and cell culture supernatants (2 pmol/200 µl) prior to the RNA isolation procedure.

### Analysis of cell growth and proliferation

For analysis of cell proliferation, cells were plated in 96-well plate and treated with dox or transfected as indicated. MTT test was performed to analyze cell growth using Cell Titer aqueous solution (Promega, #G3582). Incucyte system (Satorius, #SX5) was used to monitor cell proliferation in the duration of 3 d. Cell confluency was quantified every 20 min.

### siRNA and miRNA mimic transfection

Cells were seeded in six well plates in DMEM containing 10% FCS. Prior to transfection, the confluency was maintained at 30%. Cells were transfected with siRNA or miRNA mimics using Jetprime transfection reagent (Polyplus, #114–15) according to manufacturer’s instructions. Sequences were listed (siATG5:5′-AUUCCAUGAGUUUCCGAUUGAUGGC-3′,5′AUCCCAU.

CCAGAGUUGCUUGUGAUC-3′; siATG7: 5′-GCUGGAUGAAGCUCCCAAGGACAUU-3′,5′-CCAAGGAUGGUGAACCUCAGUGAAU-3′; mimic miR-198: GGUCCAGAGGGGAGAUAGG.

UUC; mimic miR-29a: ACUGAUUUCUUUUGGUGUUCAG).

After transfection, cells were incubated with DMEM containing 10% FCS for 24 h and treated as indicated. Cells were lysed for RNA isolation using TRIZOL method or in RIPA buffer for protein analysis.

### Generation of p62 deletion constructs and site-directed mutagenesis

PCR was carried out using template plasmid DNA and each primer in site-directed mutagenesis kit (NEB, #E0554), according to the manufacturer instructions. Briefly, PCR was performed to generate the mutant plasmid. After digestion by KLD enzymes, it was transformed into competent *E.coli* cells. Antibiotic selected colonies were picked and plasmids were extracted using miniprep purification method. All miR-198 or p62 mutant plasmids were sequenced by Sanger method and aligned by CLC sequence viewer v6.0.

### Dual reporter luciferase assays

The psiCheck™-2 plasmid (Promega, #C8021) harbors two expression cassette, driving expression of the Renilla and Firefly luciferase enzymes. Two miiR-198-binding domains were cloned into the multicloning site (MCS) located between Renilla luciferase gene and poly A tail. Firefly luciferase expression serves as an internal transduction control.

HSC-T6 cells were plated in 12-well plates 1 day before transfection. After sensor plasmid transfection, cells were incubated for 6 h and medium was changed to DMEM without FBS. Simultaneously, isolated vesicles were added to the growth medium for another 48 incubation. Cells were lysed and Renilla and Firefly luciferase signals were analyzed by dual luciferase reporter assay kit (Promega, #E1910) and luminescence microplate reader (Berthold Technologies, #LB960) according to manufacturer instructions.

### Immunofluorescene staining and immunohistochemistry

Cells were seeded on glass coverslips the day before cells were either treated with dox or transiently transfected with siRNA or plasmids. 24 h after treatment or transfection, cells were washed three times with PBS, fixed with methanol and permeabilized with 0.1% Triton X-100 in PBS for 10 min. After three washing steps with PBS, cells were blocked with 5% gelatin in PBS for 1 h at room temperature (RT). Cells were then stained with primary antibody in blocking solution at 4 °C overnight, followed by three washes with PBS. Cells were incubated with Alexa Fluor 594- or 488- conjugated secondary antibody at RT for 1 h and mounted with Mowiol medium containing DAPI (ROTH, #HP20.1). Cells were viewed under a confocal fluorescence microscope (Zeiss, #Meta 710).

Serial sections of FFPE liver biopsies from HCC patients were applied to p62 immunocytochemistry using p62 antibody(Santa Cruz, #sc-2438) immunodetection was developed using peroxidase-polymer conjugated secondary antibodies and diaminobenzidine as substrate. The stained tissue were scanned by Hamamatsu NanoZoomer Digital Pathology system.

### Combined fluorescence in situ hybridization (FISH) with immunofluorescent labeling

Cells were fixed by 4% PFA for 10 min were permeabilized by acetylation buffer (0.06 N HCl, 1.34% triethanolamine, 0.6% acetic anhydride). For prehybridization, hybridization buffer (Dako, #S1801) was incubated on the slides for 30 min at 55 °C. For in situ hybridization, 30 nM 6-FAM conjugated LNA miR-198 antisense was diluted in hybridization buffer (Dako, #S1801) used to hybridize with miR-198 at 55 °C for 1 h as previously described [20]. 6-FAM conjugated scRNA (30 nM) was used as control. After two washing steps, cells were blocked by 5% gelatin and incubated with p62 antibody (Abcam, #ab155686) for 4 h and further detected by Alexa-594 secondary antibody as described above.

### RNA immunoprecipitation (RIP)

Cells were harvested in lysis buffer (25 mM Tris (pH 8), 150 mM NaCl, 5 mM EDTA and 0.5% NP-40) supplemented with protease inhibitor cocktails (Sigma, #4,693,159,001) and 1 mM PMSF (Sigma, #329–98-6) and washed with ice cold PBS. For antibody conjugation, antibodies were coupled to Dynabeads by head-to-head rotations at RT, according to the manufacturer’s instructions. Cell lysates were centrifuged for 15 min under 4 °C at 14,000 × *g*. The transparent supernatants were collected and protein concentration were determined using BCA protein assay kit (Thermo Fischer, #23,227). 300 μg of cell lysates were pre-cleared for 1 h with end-over-end rotation with 3 μg rabbit IgG (Cell Signaling Technology, #2729) conjugated protein G coupled Dynabeads (Thermo Fischer, #10003D) at 4 °C. Samples were placed on a magnet stone, and the supernatants were collected and subsequently incubated with antibody conjugated Dynabeads on the rotator at 4 °C for 4 h. After 4 h incubation, the samples were placed on the magnet, washed three times with lysis buffer and another three times with ice cold PBS. Samples were collected and directly mixed with TRIZOL for RNA isolation.

### Western blot analysis

SDS–polyacrylamide gel electrophoresis (SDS-PAGE) was performed using the Bio-Rad Mini protein gel system. Briefly, protein samples were mixed with 4 × Laemmli buffer (Biorad, #1,610,747), denatured and directly loaded onto the gels. After electrophoresis and transfer, the membranes were then incubated in a blocking solution. After incubation with first and secondary antibodies, membranes were developed using Pierce™ ECL Western Blotting Substrate (Thermo Scientific, #32,109) according to the manufacturer’s instructions.

### Vesicle preparations, negative staining and electron microscopy

Conditioned medium was collected from cell culture. After serial centrifugation under 500 × *g*, 3000 × *g*, 12,000 × *g*, medium was filtered through 0.8 µm membrane. EVs were isolated by ultracentrifugation (Beckmann, #L8M) under 4 °C, 100,000 × *g* for 14 h. After PBS washing, vesicle pellet were resuspended in PBS for immunoblotting and RNA isolation.

Alternatively, the prefiltered medium was subject to EV isolation using exoEasy Maxi kit (Qiagen, #76,064) following the manufacturer’s instructions. The isolated vesicles were subsequently used for NTA vesicle tracking, miRNA analysis and negative staining following electron microscope imaging.

20 µl of vesicle suspension were placed on carbon-coated grids and blotted on filter paper after washing with an aqueous solution of 2% uranyl acetate. Stained vesicles were viewed using electron microscope (Zeiss, #EM902A).

### Statistical analysis

Statistical analysis was performed using SPSS software 17. Bar graphs show means ± SEM. Student’s *t* test was used. *P* values are indicated in the figure legends, *p* < 0.05 was considered statistically significant. GraphPad Prism v9.0 were used to create plots. Images were processed by Image J and Las X. Biorender (www.biorender.com) was used to illustrate signaling pathways in graphic abstract.

## Results

### miR-198 is preferentially secreted into supernatant via extracellular vesicles (EVs)

**Fig. 1:**
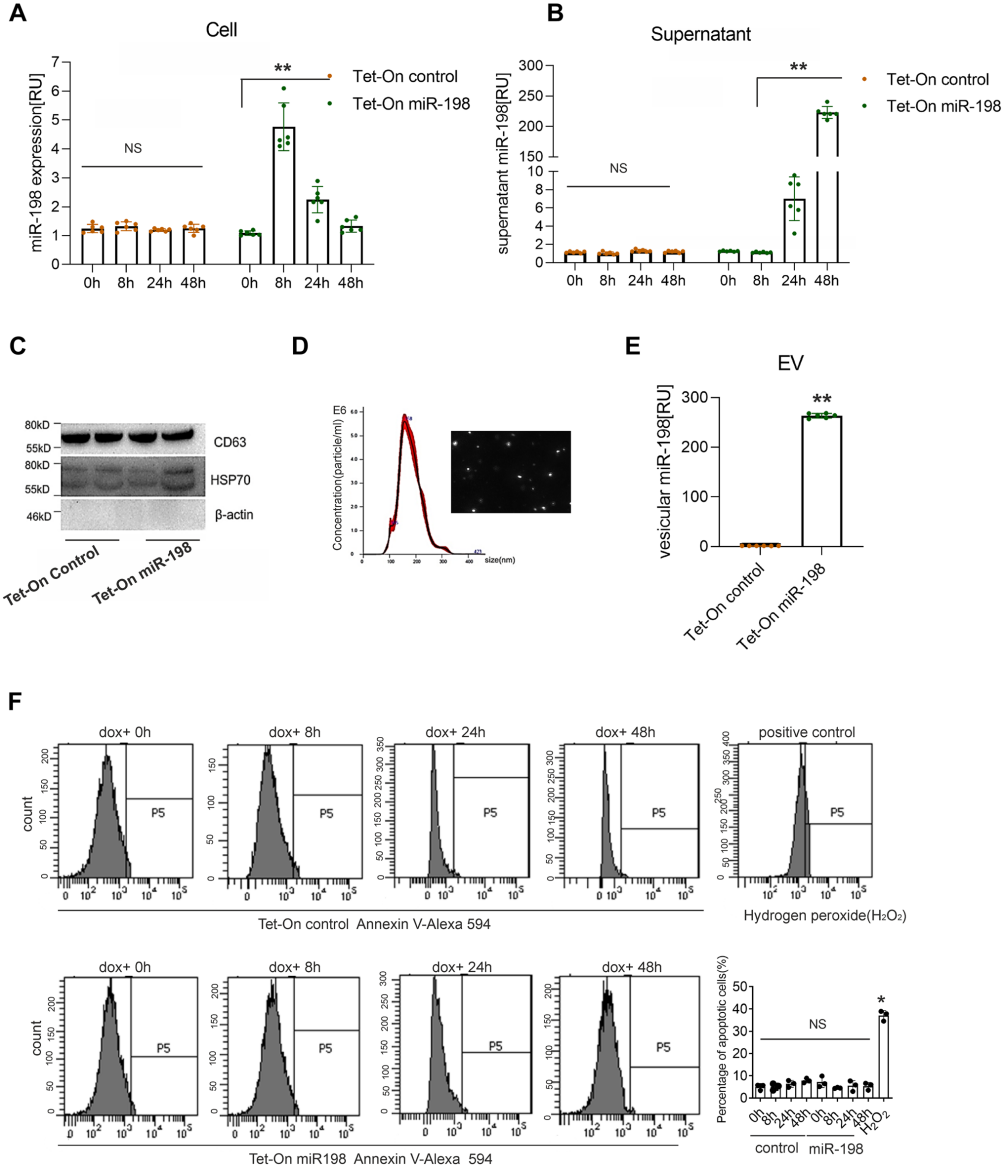
miR-198 is preferentially secreted into supernatant via exosomes. Tet-On control and Tet-On miR-198 stable HuH-7 cells treated with doxycycline (dox) for 0 h, 8 h, 24 h, 48 h, respectively. Both cells and supernatants were collected and RNA was isolated. Supernatants (48 h dox treatment) were centrifuged to eliminate dead cells and cell debris and subjected to vesicle isolation by affinity column method. The isolated vesicles were treated by RNase A, then RNase was blocked by 4 M guanidinium thiocyanate which is included in TRIZOL for RNA isolation. RNA was isolated from cells, supernatants and vesicles. Cellular (**A**), supernatant (**B**) and vesicular (**C**) miR-198 levels were analyzed by qPCR. Isolated vesicles were further characterized by immunoblotting (**D**), Nanoparticle tracking (NTA) (E). ** means *p* value < 0.001. **F** Tet-On control and miR-198 stable HuH-7 cells were treated with dox for 0, 8, 24, 48 h. Cells were harvested and stained by Annexin-V- FITC conjugate (Thermo Fischer, #A13199). Cellular apoptosis was analyzed by flow cytometry (FACS ARIA III, BD Biosciences). Here hydrogen peroxide treated HuH-7 cells were used as positive control

To study the miR-198 dysregulation in HCC [12, 13, 21], we established a stable Tet-On inducible miR-198 expression system in hepatoma cell lines, HuH-7 and Hep3B. In Tet-On miR-198 cells, miR-198 expression is induced by doxycycline (dox) treatment. We analyzed the miR-198 expression levels at different time intervals. Interestingly, the robust increase of cellular miR-198 expression was followed by a rapid decrease (Fig. 1A). Noteworthy, the time course analysis of cellular and extracellular miR-198 levels revealed the cellular decrease was accompanied by a prominent increase in the supernatant (Fig. 1B), indicating that miR-198 levels are released from liver cancer cells. Since miR-198 is a crucial tumor suppressor miRNA, we hypothesized that in response to high transgenic expression other tumor suppressor miRNAs will also be immediately eliminated via their secretion by liver cancer cells. Therefore, we stably overexpressed the tumor suppressors miR-29a and miR-145 in comparison to the miR-21, which is a main oncogenic miRNA, promoting cancer progression. In supplemental figure S1A B, we show that stable overexpression of miRNAs in hepatoma HuH-7 cells did neither result in autophagy activation nor vesicular secretion of both oncogenic miR-21 and tumor suppressor miRNAs.

Since miRNA presents universally in vesicles secreted from cells [22], we postulated miR-198 was released via EV. At first, we collected conditioned medium from Tet-On miR-198 cells. Next, EVs were isolated by column-affinity method. The vesicular fractions were characterized by immunoblotting using antibodies that recognize the EV marker proteins, CD63, TSG101 (data not shown), and HSP70, and as a negative marker β-actin was used (Fig. 1C). Nanoparticle tracking analysis (NTA) (Fig. 1D) identified the EV size between 100 and 200 nm. Quantitative PCR (qPCR), analyzing the vesicular fraction, showed more than 50-fold increase of miR-198 release from Tet-On miR-198 HuH-7 cells (Fig. 1E). Consistently, this EV accumulation was also found from Hep3B cells (Fig. S1 C D). We have calculated the copy numbers of the vesicular miR-198 secreted from cells and found that around ~ fourfold of the overexpressed miR-198 were released into EVs (data not shown). The analysis of supernatant miR-198, comprising vesicular and soluble levels, showed more than 95% of the released miR-198 are enclosed in EVs (Fig. S1 E). These data showed miR-198 decrease was mainly due to vesicle release.

Because miR-198 is a prominent tumor suppressor [13, 21], we analyzed the apoptotic effects by Annexin V staining. Stable Tet-On miR-198 HuH-7 cells were at first treated with dox for 0 h, 8 h, 24 h, and 48 h, stained with Alexa 594 fluorochrome conjugated Annexin V and subsequently analyzed by flow cytometry. Here, we observed no obvious change of apoptotic effects by miR-198 increase (Fig. 1F). Additionally, we ensured that miR-198 levels in transgenic Tet-On stable cell systems did not exceed the physiological expression of liver parenchymal cells obtained from a healthy liver (Fig. S1F). Therefore, our miR-198 Tet-On expression system mimics endogenous expression under physiological conditions.

Hence, miR-198 in liver cancer cells shows a preference to be packaged in EVs and released into extracellular space.

### miR-198 enhances autophagic activity in liver cancer cells

**Fig. 2:**
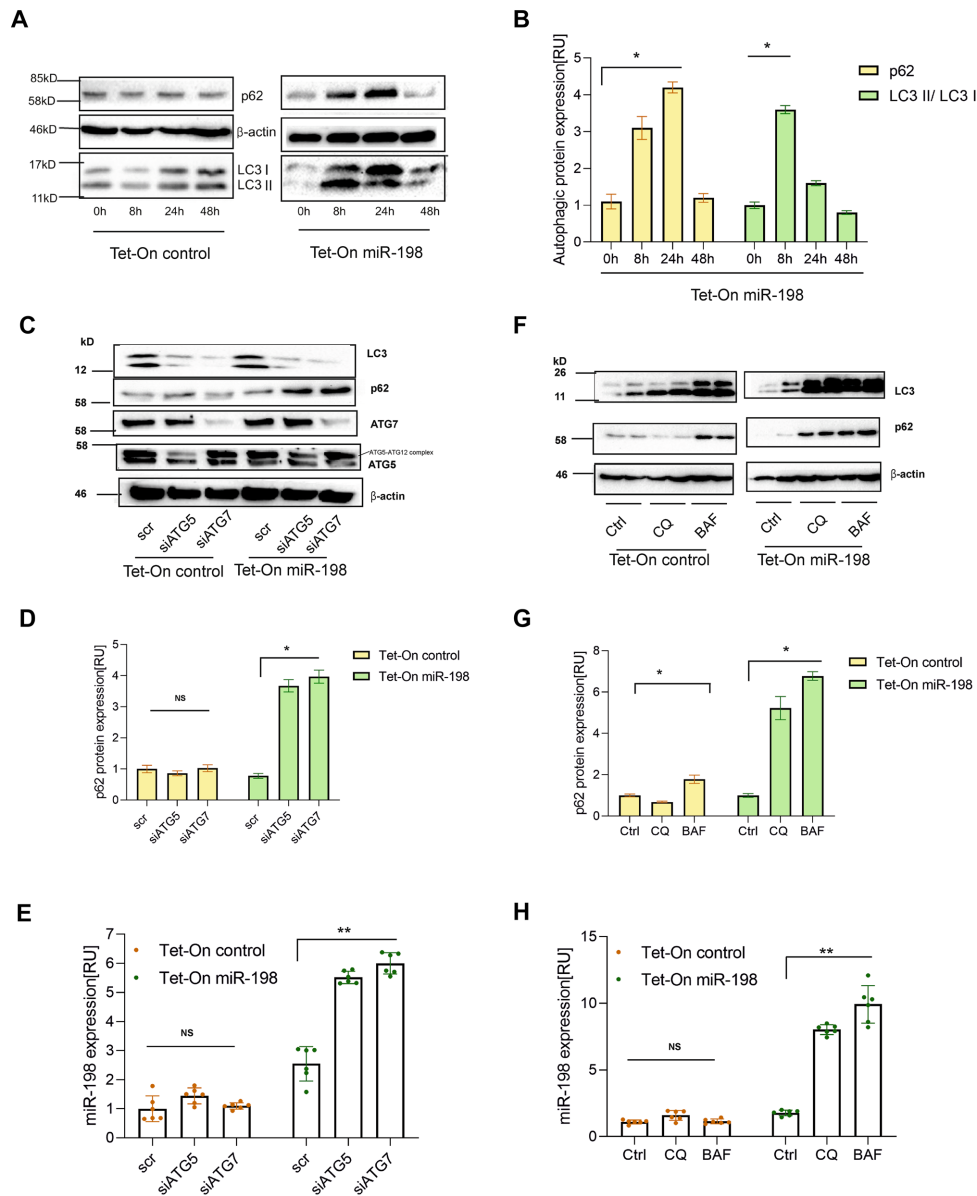
Increased miR-198 expression enhances autophagic activity. Tet-On control and miR-198 stable HuH-7 cells were treated with dox for 0, 8, 24, 48 h. Cells were harvested and cell lysates were subject to immunoblotting for the expression of the autophagy marker p62 and LC3 protein expression (**A**). Quantitative analysis of p62 and LC3 protein expression of Tet-On miR-198 stable HuH-7 cells were performed using Image J (**B**). Furthermore, cells were at first transfected with scRNA, siATG5 or siATG7. 24 h post-transfection, cells were treated with dox for another 24 h. Immunoblotting was performed to analyze p62, LC3, ATG5 and ATG7 protein expression (**C**), and statistical analysis of p62 protein expression by image J (**D**), miR-198 expression was analyzed by qPCR (**E**). Moreover, cells were treated with dox for 8 h and then subject to BAF or CQ for another 16 h. Cells were harvested for both RNA isolation and protein analysis. miR-198 expression was analyzed by qPCR (**F**), p62 and LC3 protein expression by immunoblotting (**G**) and statistical analysis of p62 protein expression by image J (**H**). * means *p* value < 0.05, **p < 0.001

Previous studies have correlated autophagic process to vesicle secretion [2, 23] and autophagy contributes to the secretion of IL-1β [24]. To analyze autophagic effects by miR-198, we performed immunoblotting using antibodies against autophagy marker proteins, LC3 and p62. Corresponding to cellular miR-198 ‘increase and decrease’ pattern (Fig. 1A,B), we observed elevated expression of both proteins, p62 and LC3, in particular of the mature, autophagosome membrane bound LC3 II form in the first 8 h in response to dox-induced miR-198 expression (Fig. 2A,B). Likewise, high p62 and LC3 expression levels were also tuned back to its original levels at 48 h (Fig. 2A,B), implicating a correlation between miR-198 and autophagy.

To investigate this correlation, we silenced ATG5 and ATG7 expression by RNAi to impede autophagic activity. siRNA-mediated knock-down was validated at both transcriptional levels (Fig. S2A,B) and protein levels (Fig. 2C). Interestingly, ATG5 KD and ATG7 KD led to not only 5–sixfold increase of cellular miR-198 (Fig. 2E) but also more than 50% reduction of both EV amount (Fig. S2 C) and vesicular miR-198 level (Fig. S2 D). Since ATG5 and ATG7 disrupts the synthesis of LC3 protein [25], a strong decrease of LC3 protein expression was found (Fig. 2C). Importantly, p62 protein expression was higher in miR-198 expressing cells compared with non-overexpressed cells (Fig. 2C,D). These data implicate that miR-198 enhances autophagy and reversely autophagy contributes miR-198 secretion.

To validate miR-198 enhances autophagy, we used two autophagy inhibitors, chloroquine (CQ) and bafilomycin A1 (BAF), preventing the maturation of autophagosome and p62 disposal [26]. We validated the autophagy inhibition by an increased expression of both LC3 and p62 proteins (Fig. 2F,G). Corresponding to ATG5/7 KD, BAF and CQ treatment caused miR-198 accumulation in Tet-On miR-198 cells (Fig. 2H), confirming autophagy leads to miR-198 reduction. To note, in miR-198 expressing cells, BAF raised p62 protein levels by ~ sevenfold, whereas in the non-expressed cells only ~ threefold was found (Fig. 2F,G). Furthermore, we repeated the treatment of the Tet-On control and miR-198 cells with dox and BAF in Hep3B cells, and validated the increased expression of LC3 and p62 protein by miR-198 (Fig. S2E), confirming an enhanced autophagic flux triggered by miR-198. These data reinforce miR-198 induces autophagy and autophagy in turn reduces miR-198 level.

### miR-198 is recruited to autophagosome-derived vesicular fractions

**Fig. 3:**
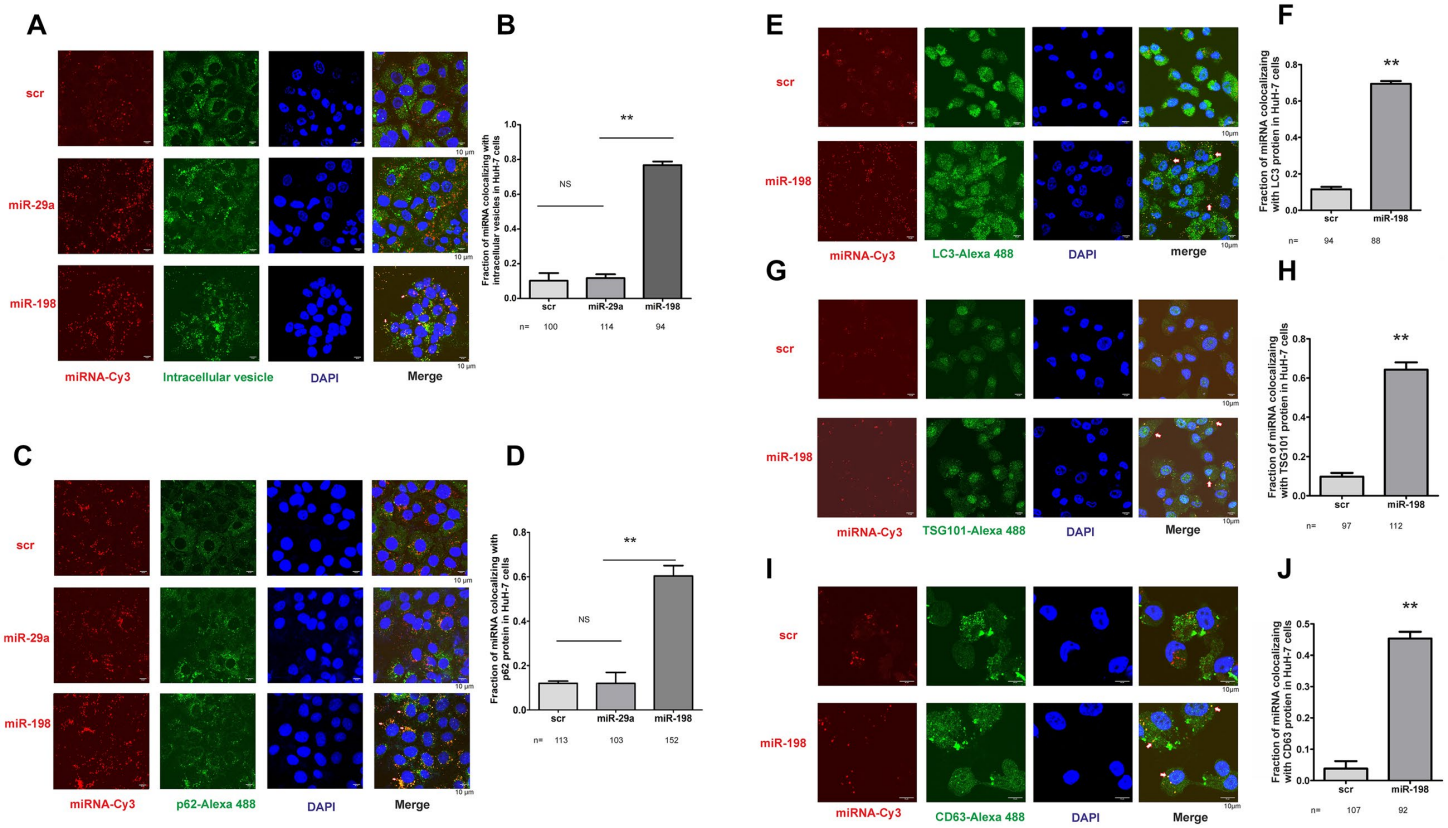
miR-198 is recruited into autophagosome-derived vesicular fraction. HuH-7 cells were transfected with Cy3 conjugated RNA oligonucleotides. 6 h after transfection, fresh medium was changed. After 24 h of incubation, cells were fixed and subjected to immunofluorescence staining to detect autophagy and EV marker proteins. For combined FISH and immunofluorescence staining, cells were at first hybridized with miR-198 antisense or scRNA probes and then immunostained. Fluorescence signals were observed under confocal microscope. The co-localization of scRNA with p62 (**A** **B**), LC3 protein (**E** **F**), TSG101 protein (**G** **H**), CD63 protein (**I** **J**) were presented. The combination of FISH using 6-FAM conjugated LNA-miR-198 antisense and immunofluorescence staining was performed using Tet-On miR-198 stable cells after dox treatment for 8 h. The co-localization of miR-198 with p62 protein was shown (**C D**) as microscopic images and the statistical analysis of co-localization was analyzed by Image J. Blue, DNA; Red, miR-198; Green, intracellular vesicle, p62, LC3, TSG101, CD63 protein as indicated. Scale bar: 10 µm. ** means *p* value < 0.001

That we have discovered miR-198 is secreted into EVs and enhances autophagy, prompted us to hypothesize miR-198 is enclosed in autophagosomes. We used Cy3 conjugated miR-198 mimic to study intracellular tracking and EV loading. To disrupt the entrapment of miRNA mimic in endosome, we used a polyethylenimine-based transfection reagent [27], which belongs to the cationic polymers. Cationic polymers induce proton sponge mechanism leading to efficient si/miRNA endosomal escape [28–31]. Indeed, after staining of the cellular vesicles by lipophilic carbocyanine dye, we observed no co-localization of scramble (sc) RNA and miR-29a with intracellular vesicles, validating the endosomal escape and efficient cytoplasmic release (Fig. S3 A). Subsequently, we used miR-198-Cy3 mimic, performed vesicle staining and immunochemistry using antibodies against autophagic proteins. Strikingly, we observed strong co-localization of miR-198 with both intracellular vesicles (Fig. S3A) and p62 protein (Fig. 3A,B), whereas no such co-localization was found for scRNA and miR-29a. It indicates that miR-198 is recruited into p62 protein-related vesicle fractions.

To confirm the recruitment into p62 protein vesicular fractions, we performed in situ hybridization (ISH) combined with p62 immunostaining to simultaneously detect endogenous miR-198 and p62 protein. MiR-198 was detected by its antisense LNA probes and p62 protein labeled by its antibody. Consistently, we detected strong co-localization of p62 protein and miR-198 (Fig. 3C,D). As an autophagosome membrane marker, LC3 protein was also found to co-localize with miR-198 (Fig. 3E,F), suggesting miR-198 is encapsulated into autophagosome-derived vesicular fractions.

To validate miR-198 in the vesicular fractions, we utilized antibodies against vesicular marker proteins, TSG101 and CD63 [32]. Interestingly, the same co-localization was observed (Fig. 3G–J). Therefore, our data illustrate the recruitment of miR-198 into autophagosome-derived vesicular fractions.

### miR-198 is selectively loaded by p62 protein into vesicle fractions

**Fig. 4:**
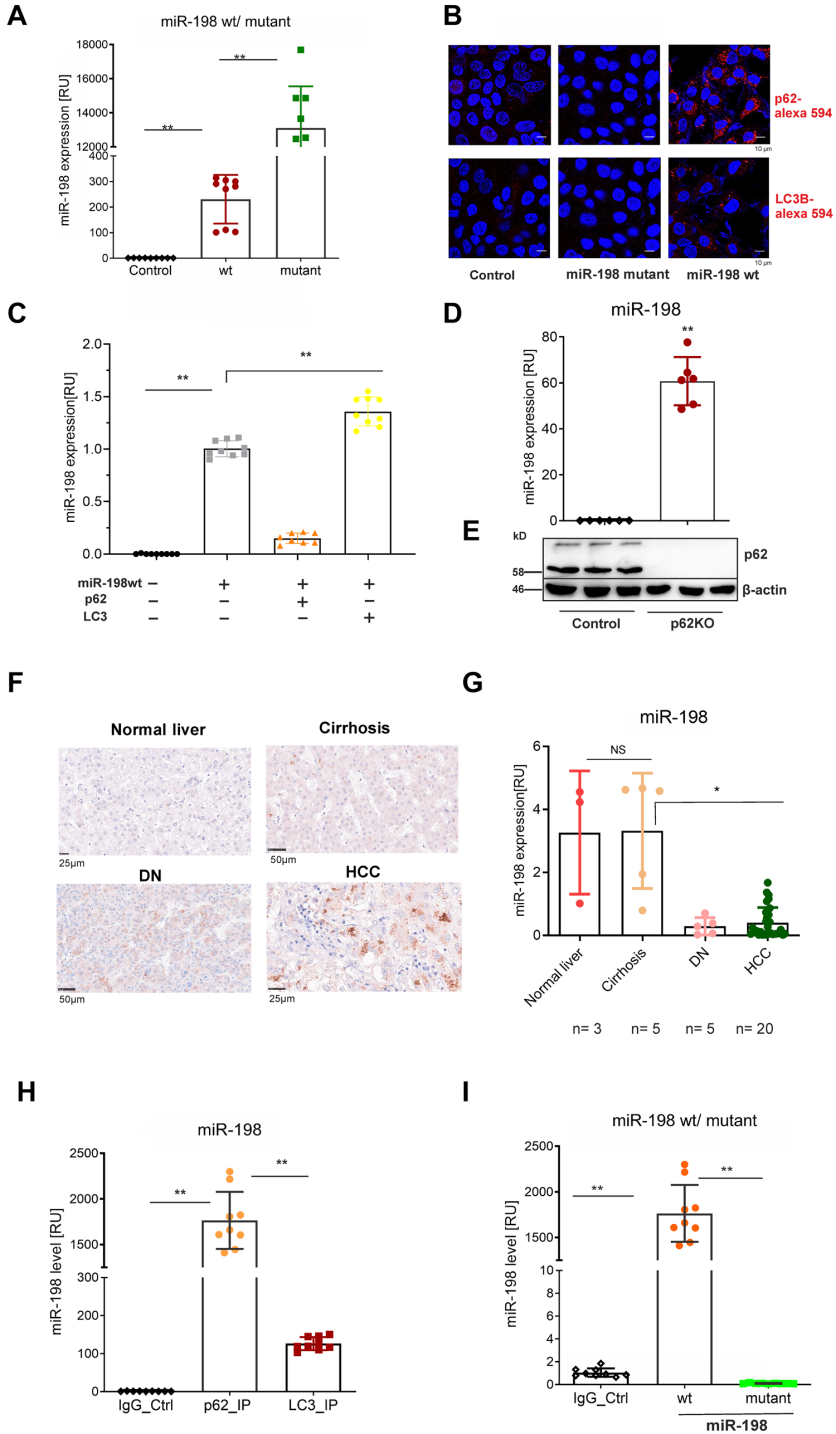
miR-198 is selectively loaded by p62 protein. HuH-7 cells were stably transfected with plasmid encoding wt or mutant miR-198 expression under CMV promoter. The cells were lysed for RNA isolation and cellular miR-198 expression (**A**) was analyzed by qPCR. In parallel, cells were fixed for immunostaining to detect LC3 or p62 protein (**B**). Blue, DNA; Red, LC3 or p62 as indicated. Scale bar: 10 µm. HuH-7 cells were at first transfected with plasmid encoding miR-198 under CMV promoter. 24 h after transfection, cells were trypsinized and subject a second transfection using the plasmid encoding either p62 or LC3 protein expression. 24 h after the second transfection, cells were harvested for RNA isolation, miRNA expression (**C**) was analyzed by qPCR. HuH-7.^P62KO^ monoclone cells were harvested for RNA isolation and protein lysates. The endogenous miR-198 expression (**D**) was analyzed by qPCR, p62 protein expression (**E**) by immunoblotting. P62 protein expression (**F**) was detected by immunohistochemistry and miR-198 expression (**G**) by qPCR in human biopsies with liver cirrhosis (*n* = 5), dysplastic nodules (*n* = 5), HCC (*n* = 20). As controls, healthy livers (*n* = 3) were used. Cells were transfected with plasmids encoding miR-198 wt or mutants under CMV promoter, 24 h after transfection, cells were lysed for RIP-qPCR to detect miR-198 wt (**H**) bound with LC3 or p62 protein. In parallel, p62 bound miR-198 wt and mutant (**I**) levels were also measured. ** means *p* value < 0.001

Since the co-localization with vesicular proteins was only found for miR-198, we postulated that miR-198 was selectively recruited to vesicle fractions. At first, we performed site-directed mutagenesis to mutate the seed region of miR-198, changing the miR-198 wildtype sequence GGUCCAGAGGGGAGAUAGG to the miR-198 mutant GG*AAUUC*AGGGGAGAUAGG. In comparison with wild type, however, we detected higher cellular levels of mutants under the same conditions (Fig. 4A); but the autophagic proteins (LC3 and p62) were not increased (Fig. 4B), indicating high level of mutants doesn’t enhance autophagy. This data points to the selective induction of autophagic effects by miR-198.

In the next step, we investigated which autophagic protein decreases miR-198 level. At first, we analyzed the most important autophagic proteins, p62 and LC3. Surprisingly, we found p62 decreased nearly 90% of miR-198 level, whereas LC3 protein did not (Fig. 4C). Interestingly, the same effect was also found in non-tumor HEK293 cells (Fig. S3 B), revealing an important role of p62 protein in miR-198 decrease. Subsequently, we knocked out p62 genetic locus in HuH-7 by the Crispr/Cas9 technology. The lack of p62 protein was confirmed at protein level by immunoblotting (Fig. 4D). Notably, we detected more than 60-fold increase of endogenous miR-198 levels after p62 KO (Fig. 4D). Therefore, we identified p62 protein as essential repressor of miR-198 level; specifically, an inverse correlation between p62 and miR-198 expression in vitro was found.

Furthermore, we attempted to validate this correlation in vivo. Because miR-198 expression is primate specific [33], formalin-fixed and paraffin-embedded (FFPE) liver tissues were collected from patients at different stages of liver diseases including cirrhosis, dysplastic nodules and HCC. We performed p62 protein-based immunohistochemistry and analyzed the corresponding miR-198 expression by qPCR. In agreement with previous study [34], p62 protein expression was enormously upregulated in HCC (Fig. 4F), however, the miR-198 expression was reduced by nearly 90% compared to healthy controls (Fig. 4G). Therefore, we identified the inverse correlation between miR-198 and p62 protein expression in vivo.

It is plausible that p62 protein represses miR-198 level by EV secretion. To investigate the repressive effect on miR-198, we isolated EVs from p62KO cells and analyzed the vesicular miR-198 release. Remarkably, p62 KO strongly decreased nearly 50% of the amount of released EVs (Fig. S3C) and led to more than 90% reduction of miR-198 secretion (Fig. S3D). These results show that p62 protein contributes to vesicle secretion to reduce miR-198 expression level.

As p62 protein dissipates miR-198 by vesicle secretion, we hypothesized if p62 loads miR-198 through a protein-RNA interaction manner. Following this idea, we analyzed RNA–protein interaction by RNA immunoprecipitation followed by quantitative PCR (RIP-qPCR); strikingly, we found an immense amount of miR-198 molecules bound to p62 proteins (Fig. 4H). LC3 protein is an interacting partner of p62 protein [7] as validated by co-immunoprecipitation (Fig. S4 A). RIP-qPCR showed that less than 10% of p62 bound miR-198 were detected in the LC3 precipitates (Fig. 4H). Importantly, we confirmed the selective binding of p62 protein to miRNAs by expressing miR-29a, miR-21 and miR-198 mutant under the same conditions. Here, none of the significant enrichments were found (Fig. 4I and Fig. S4B). That corresponds to the finding that p62 protein fails to reduce both miR-21 and miR-29a levels (Fig. S4 C D). Moreover, the selective interaction of p62 with miR-198 could also be seen from p62 immunochemistry, showing strong co-localization for miR-198 but not for scRNA and miR-29a (Fig. 3C,D). Taken together, it reveals that p62 protein selectively loads miR-198 into autophagosome-derived vesicular fractions.

### p62/miR-198 interaction is conjointly regulated by different functional domains

**Fig. 5:**
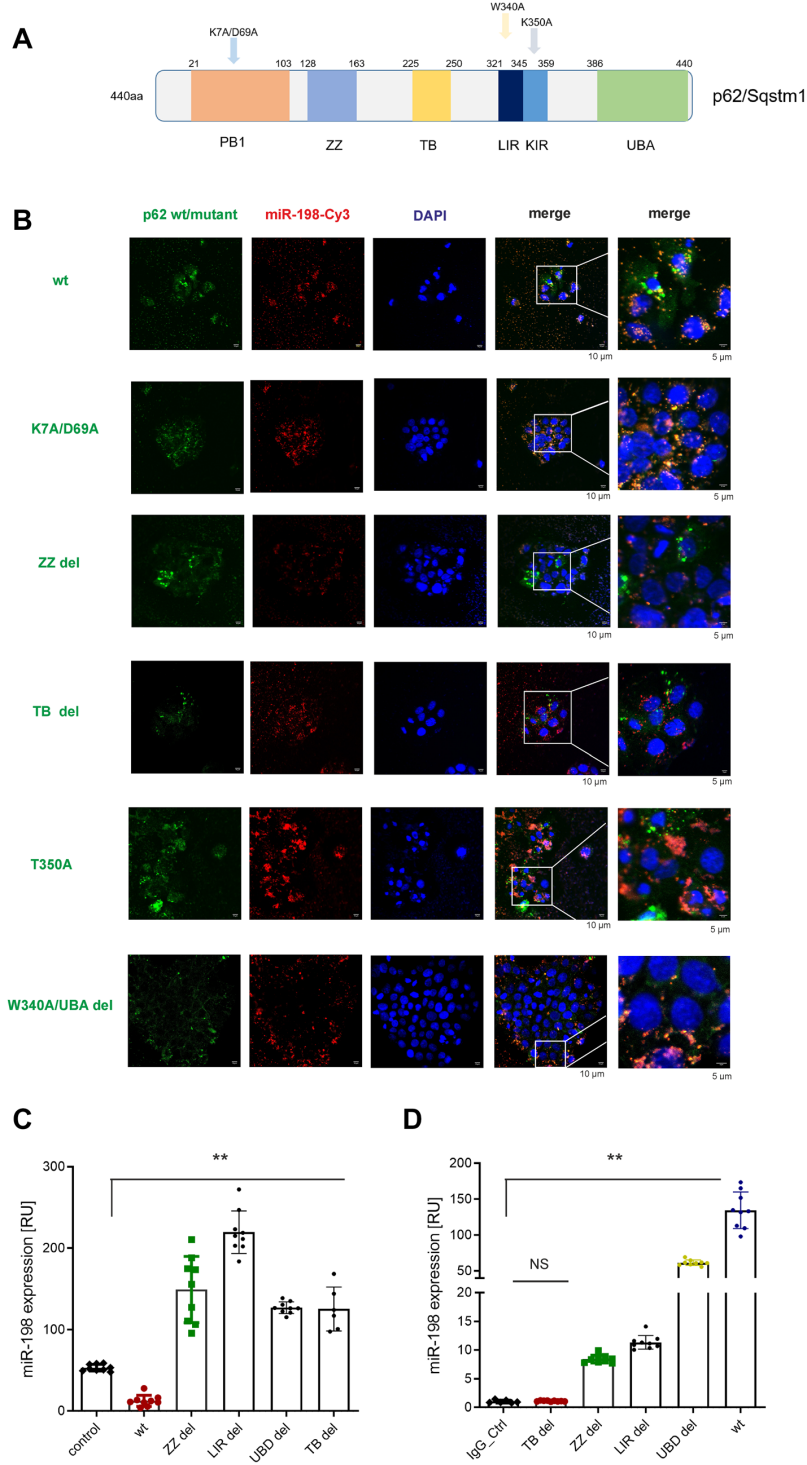
P62 / miR-198 interaction is conjointly regulated by different functional domains. **A** Schematic presentation of functional domains of p62 protein. PB1: Phox and Bem 1 domain; ZZ: zinc finger domain; TB: TRAF6 binding domain; LIR: LC3 interaction region; KIR: Keap1 interaction region; UBA: ubiquitin associated domain. HuH-7 ^p62 KO^ cells were transiently co-transfected using miR-198-Cy3 and the plasmids expressing different p62 variants. 24 h after transfection, cells were fixed and immunostained using p62 antibody. The co-localization of miR-198-Cy3 with p62^WT^, p62^K7A/D69A^, p62^ZZ del^, p62^TB del^,p62^T350A^, p62.^W340A/UBA del^ (**B**) variant were viewed under confocal microscope. In parallel, cells were lysed for RNA isolation or RIP-PCR using either IgG or p62 antibody. Cellular miR-198 expression (**C**) and p62 protein variants bound miR-198 (**D**) were detected by qPCR. Red: miR-198; Green: p62 variant as indicated; blue: DNA stained by DAPI. Scale bars 10 µm or 5 µm as indicated. NS: no statistical significance. ** means *p* value < 0.001

As a scaffold protein, p62 has different functional domains, through which p62 binds to its interaction partners and regulates cell metabolism. These domains were depicted in (Fig. 5A). By site-directed mutagenesis, we have constructed various p62 truncated variants, including deletion of Zinc finger domain (ZZ), TRAF6 binding domain (TB), Ubiquitin-associated domain (UBA), as well as point mutations, K7A/D69A of Phex Bem1p domain (PB1) [35], W340A of LC3 interaction region (LIR) [35] and T350A of Keap1 interaction domain (KIR) [36].

PB1 domain drives the self-oligomerization, p62 protein utilizes its UBA domain to interact with (poly)ubiquitinated protein complex, TB domain with TRAF6 protein, KIR region with Keap1 protein, LIR region with LC3 protein. To study the functional roles of different domains of p62 protein, we constructed expression vectors expressing p62 mutant variants by site-directed mutagenesis. Neither point mutation nor deletion of PB1 domain did affect p62 protein binding with miR-198 (Fig. S5A). Importantly, we found impaired co-localization of miR-198 and the p62 protein mutants (including p62^UBA del^, p62^LIR mu^, p62^KIR del^ p62^TB del^ and p62^ZZ del^) (Fig. 5B and S5A), and the lowest co-localization was shown for p62 ^TB del^ (Fig. S3D), suggesting TB domain might be an indispensable region for miR-198 interaction.

Moreover, miR-198 binding with different p62 mutant variants was studied by RIP-PCR. Here, we observed increased cellular miR-198 levels after LIR and UBA domain deletion (Fig. 5C), p62 ^LIR del^ and p62 ^UBA del^ protein were still bound with miR-198 albeit their co-localization was mildly decreased (Fig. 5D). However, p62 ^TB del^ protein overexpression elevated miR-198 expression (Fig. 5C) but we did not detect any miR-198 in the precipitated protein compound (Fig. 5D).

These data indicate that miR-198 interaction with p62 protein are collectively dependent on the integrity of the functional domains.

### The p62 protein loads miR-198 into secreted EVs

We have discovered p62 protein dissipates miR-198 into extracellular space, however, p62 protein level was reduced when miR-198 was secreted (Fig. 2A,B), pointing that p62 protein might be co-secreted into EVs.

**Fig. 6:**
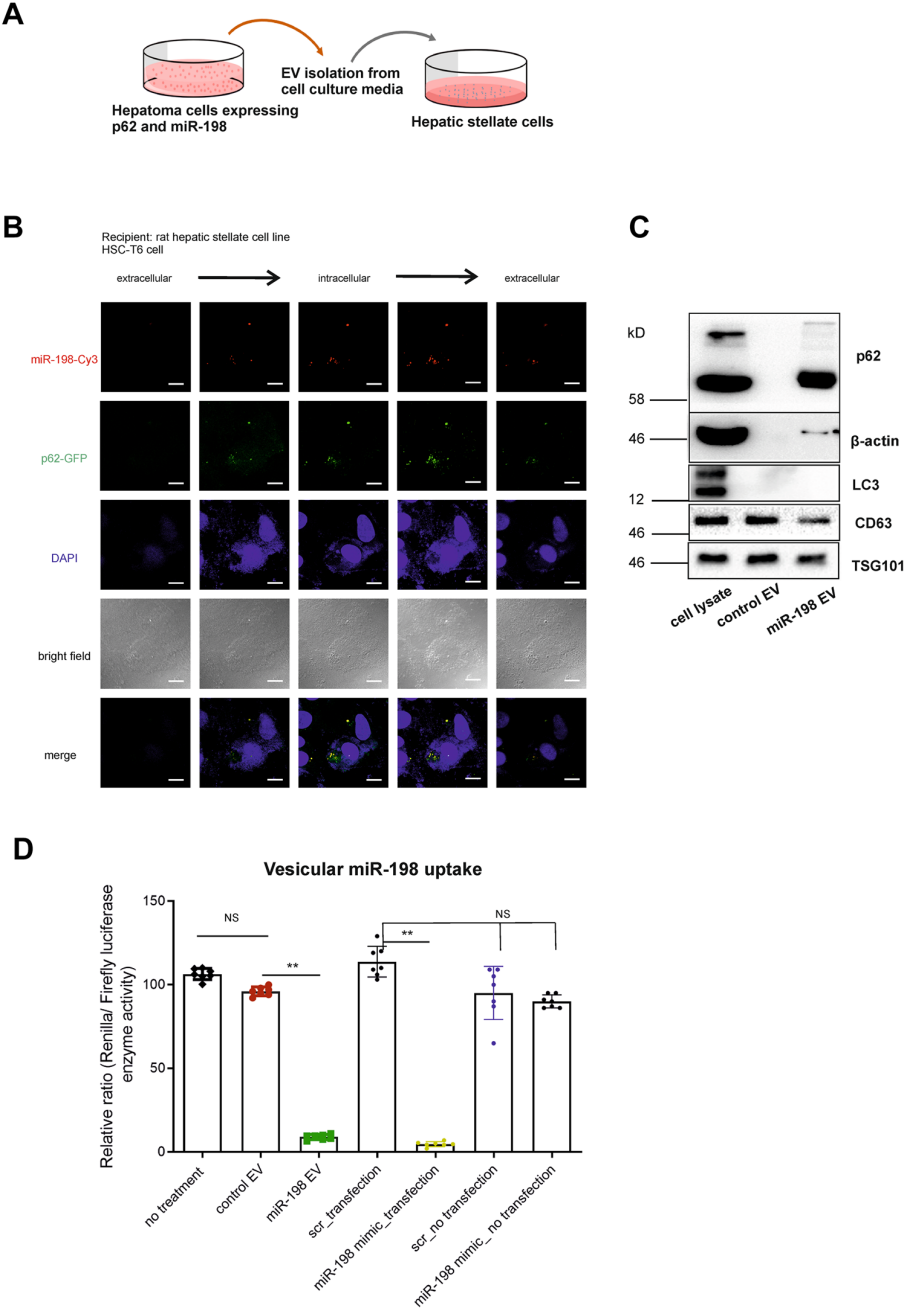
p62 loads miR-198 secretion into EV. **A** Workflow of p62 protein chaperoning miR-198 secretion in EVs: Supernatants were collected from HuH-7 cells, transiently or stably transfected with miR-198, centrifuged to eliminate dead cells and cell debris. The vesicles were isolated by ultracentrifugation. Pelleted vesicles were resuspended either in PBS for protein component analysis or in DMEM medium for vesicle uptake assays, analyzed by dual luciferase reporter assay and microscopic imaging. (**B**) Representative fluorescence confocal microscopy Z-stack images of recipient hepatic stellate cells (HSC-T6 cells). HuH-7^p62KO^ cells were co-transfected with miR-198-Cy3 and p62-GFP encoding plasmid. 24 h after transfection, medium was changed using fresh DMEM without FBS supplement and cells were further incubated for another 48 h. EVs were isolated from the conditioned medium by ultracentrifugation. The pelleted EVs were treated with RNase A and resuspended in DMEM medium before treating HSC-T6 cells. 24 h after treatment, HSC-T6 cells were fixed by 4% formaldehyde, stained with DAPI and viewed under confocal microscope. Z-stack images were acquired at different horizontal distances of the cells by confocal microscope. Blue, DNA; Red, miR-198; Green, p62 protein. Scale bar: 15 µm. **C** Immunoblotting analysis of vesicle entrapped proteins. Tet-On control and Tet-On miR-198 stable HuH-7 cells were treated with dox and incubated in DMEM medium (without FBS) for 48 h. Cell supernatant were collected and centrifuged to eliminate dead cells and cell debris. The vesicles were isolated by ultracentrifugation. Pelleted vesicles were resuspended in PBS for Western Blot analysis using the antibodies against p62, β-actin, LC3, TSG101 and CD63 protein. **D** Gene expression analysis of vesicle uptake into hepatic stellate cells (HSC-T6 cells). Vesicle preparation: Tet-On control and Tet-On miR-198 stable HuH-7 cells were treated with dox and incubated in DMEM medium (without FBS) for 48 h and cell supernatant were collected and centrifuged to eliminate dead cells and cell debris. The vesicles were isolated by ultracentrifugation. Pelleted vesicles were resuspended in PBS and subjected to RNase A treatment. Finally, the vesicles were ultracentrifuged and resuspended in DMEM for further use. Treatment of recipient cells: HSC-T6 cells were transiently transfected using miR-198 sensor plasmid. 24 h after transfection, medium was changed using vesicle- DMEM mixture (described above). Here, we used mimic miR-198 transfection into HSC-T6 cells as positive control; as negative control miR-198 mimic was directly added into the culture medium. Cells were incubated for another 24 h. Finally, cells were harvested for measurement of Renilla and Firefly luciferase enzyme activity. NS: no statistical significance. ** means *p* value < 0.001

To study p62 protein secretion during miR-198 release, we stably expressed GFP-fused p62 protein in p62 KO hepatoma cells and used Cy3 conjugated miR-198 mimics. The EVs were isolated from conditioned media, resuspended in fresh cell growth media and directly incubated with recipient cells (Fig. 6A). To best mimic the cell–cell interaction, we chose rat hepatic stellate cells (HSC-T6) as recipient cells, which is a type of liver cancer-associated fibroblasts (CAFs) and demonstrated to assign protumorigenic effects via crosstalk with cancer cells [37]. Since miR-198 is not expressed in murine species [33], no endogenous miR-198 in recipient cells (HSC-T6) would complicate the analysis of vesicular miR-198 uptake. Notably, we observed both the internalization and co-localization of p62 protein and miR-198 in recipient cells (Fig. 6B). Consistently, p62 secretion into EVs was confirmed by Western Blotting where an enormous amount of p62 protein was detected in miR-198 enriched EVs (Fig. 6C). However, LC3 protein was not secreted, indicating that the release of p62/miR-198 complex did not occur from direct expulsion of autophagosomes, rather the fusion of autophagosome with multivesicular bodies (MVB) is possible as characterized by CD63 and TSG101 proteins (Fig. 6C). Nevertheless, we have discovered p62 chaperons miR-198 secretion into EVs.

To validate the cargo sequestration in EVs, we attempted to study the function of EV transfer and focused on the changes of gene expression of recipient cells after miR-198 intake. We applied dual luciferase reporter system to detect miR-198 using a miR-198 sensor construct. To this end, miR-198-binding sequences were inserted into the 3´UTR of Renilla luciferase gene using Firefly luciferase as internal control. HSC-T6 cells were transfected with miR-198 sensor vector and subsequently treated with EVs. In consistence to mimic miR-198 transfection as positive control, we found treatment of miR-198 enriched EVs strongly inhibited Renilla luciferase reporter expression (Fig. 6D), revealing vesicular miR-198 binds with its target genes and leads to change of Renilla luciferase enzyme gene expression. Whereas direct application of naked mimic miR-198 did not cause the inhibition (Fig. 6D), ruling out the cell uptake of soluble, unpackaged miR-198. It corroborates that miR-198 is enclosed in EVs and vesicular miR-198 can be delivered to recipient cells.

Taken together, miR-198 secretion is chaperoned by p62 protein into EVs and miR-198 is still functional after transfer.

### Autophagy inhibition enhances miR-198-mediated tumor suppression

**Fig. 7:**
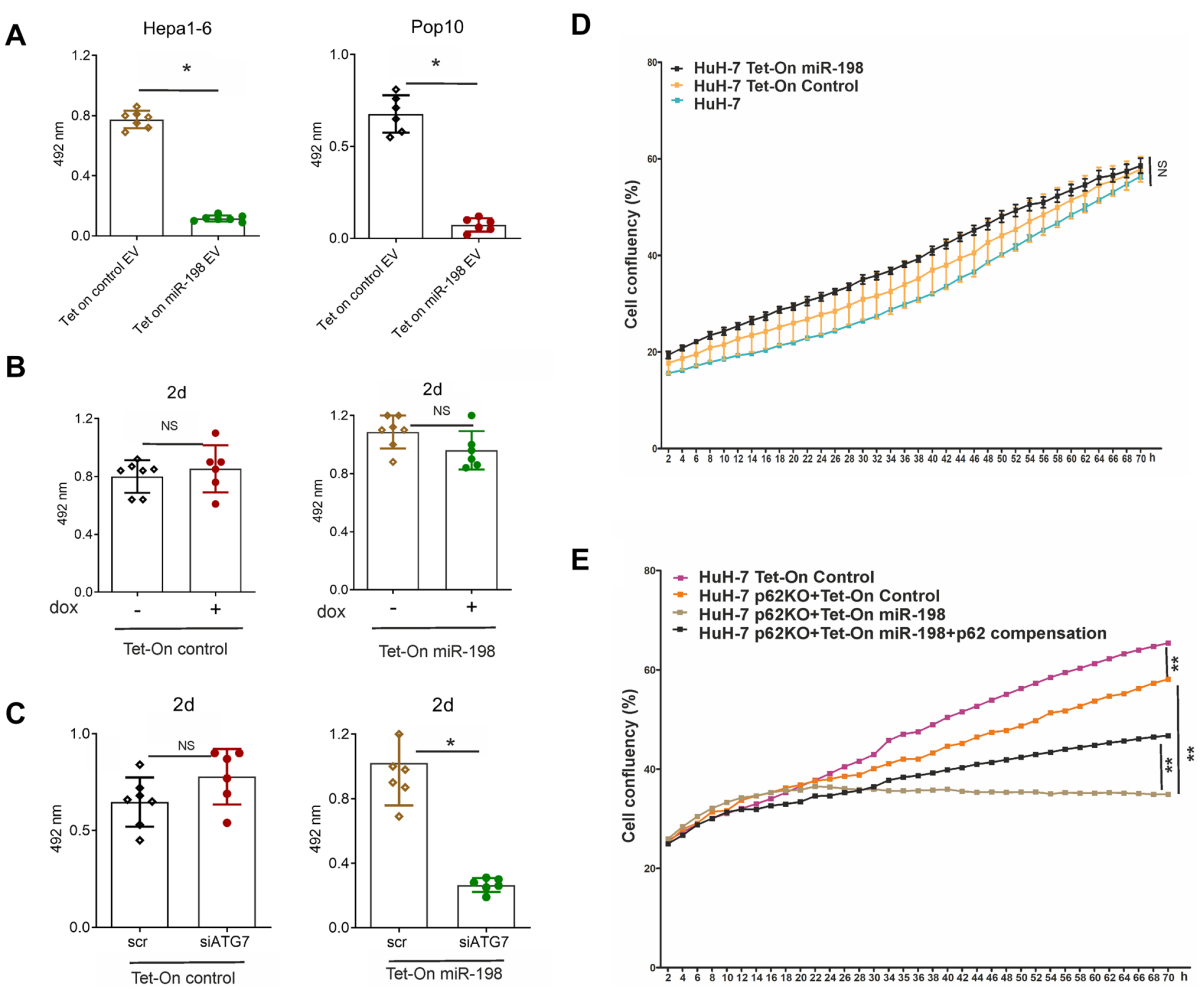
Autophagy inhibition enhances miR-198 mediated tumor suppression. The cell viability of Tet-On control (**A**) and miR-198 (**B**) stable HuH-7 cells was treated with dox for 2d, 4d and 7d and tested by the MTT assay. Cell viability was analyzed at 492 nm wavelength by Multiscan Ascent photometer. NS: no statistical significance. Tet-On control and miR-198 stable HuH-7 cells were at first transfected with scRNA or siATG7 and then treated with dox for 24 h. Cell viability (**C**) was analyzed using the same method as above. The values were presented in average ± SEM. *p < 0.05. Tet-On control and miR-198 stable HuH-7 cells were treated with dox for 3 d and cell proliferation was analyzed by Incucyte cell proliferation system (**D**). The Tet-On control and miR-198 stable HuH-7 ^p62KO^ cells were treated with dox for 3 d and analyzed by Incucyte system. For p62 compensation, cells were transfected with plasmid encoding p62 protein expression. 24 h after transfection, cells were treated with dox for 3 d and cell proliferation was analyzed by Incucyte system (**E**). The values were presented in average ± SEM. **p < 0.001, *NS*  no significance

MiR-198 is a potent tumor suppressor and inhibits cell growth [13, 21]. We analyzed the vesicular miR-198 function by treating recipient cells with EVs secreted from Tet-On stable control and miR-198 cells. Here, we observed the inhibition of more than 90% of cell growth after treatment with miR-198 EVs (Fig. 7A). However, in the donor cells that secretes miR-198, we observed neither inhibition of cell viability (Fig. 7B) nor suppressed cell migration (data not shown). Considering that autophagy downregulates miR-198 level, it is conceivable that autophagy disrupts miR-198’s function as tumor suppressor. To follow this path, we treated the cells with dox to elevate miR-198 expression and simultaneously with siATG7 to inhibit autophagy. In the mock controls, no obvious growth inhibition was found; however, we detected a strong growth inhibition of Tet-On miR-198 cells in the duration of 2 d (Fig. 7C), indicating autophagy is one barrier for miR-198 mediated growth inhibition.

To confirm the functional inhibitory role of autophagy in miR-198, we used Incucyte system to analyze cell proliferation. Real time monitoring of cell proliferation during 3 days confirmed that cell growth was not inhibited in response to dox-induced elevation of the tumor suppressor miR-198 as well as the mock control (Fig. 7D). However, in combination with p62KO, miR-198 led to a rapid cell growth stagnation (Fig. 7E). Notably, we observed partially restored cell proliferation by p62 compensation (Fig. 7E), confirming that autophagy impedes miR-198 effect on cell proliferation. Therefore, we conclude that autophagy strongly impairs miR-198 tumor suppressive function.

## Discussion

In this study, we provide compelling evidences that HCC cells control tumor suppressor miR-198 expression by autophagy-mediated vesicle release, involving the autophagy receptor protein SQSTM1/p62. Thus, we can assign a mechanism-based function to autophagy-associated EV release for dissipating tumor suppressor miR-198. Our data also show that the disruption of autophagy greatly restores cellular miR-198 levels and enhances miR-198 mediated tumor suppression, which has strong implications for miRNA therapy against HCC.

We show that miR-198 in liver cancer cells is chaperoned by p62 protein and this process is mediated by autophagy-associated EV release. Previous studies have shown p62 protein is degraded by fusion of autophagosome and lysosome [7, 8]. However, our results revealed for the first time that the autophagy, induced by miR-198, directs p62 protein into EVs, thereby providing a new avenue for cellular p62 protein disposal.

Our gain- and loss-of-function experiments demonstrate that p62 strongly represses miR-198 level. This conclusion is corroborated by the findings that miR-198 expression is elevated by p62 knockout and mutation. Noteworthy, we show for the first time that p62 protein selectively controls tumor suppressor miR-198 levels, maintaining its low cellular presence via autophagy-associated secretion. It is possible that high expression of p62 in tumor cells [34] triggers autophagy-associated vesicular miR-198 release. Indeed, we observed increased intracellular miR-198 levels, decreased vesicular miR-198 levels and lower number of released vesicles after p62 knockout; in contrast, however, LC3 protein seems to inhibit miR-198 release from HCC cells. This discrepancy coincides with their opposite outcomes in patient survival and recurrence where LC3 improves [38, 39] but p62 exacerbates it [34]. This disparity was also found in our study that LC3 protein was less than direct interaction with miR-198, compared with p62. Therefore, the autophagic secretion of miR-198 depends more on p62 than LC3.

We have found enormous miR-198 in p62 protein immunoprecipitates and identified their co-localization not only in liver cancer cells, but also in recipient cells that are treated with miR-198 enriched EVs. Since mutation of p62 protein has only impaired, but not blocked the interaction with miR-198, miR-198 might not directly interacts with p62 protein. Instead, we would envisage p62 as a ‘magnet’ to capture protein complex where miR-198 is conglomerated. Importantly, the role of p62 in complexing and sorting out superfluous miRNA seems to be miRNA-specific, because scRNA, miR-29a and the miR-198 mutant did not interact with p62 protein. Notably, sequence motif of miRNA was shown to determine its sorting into exosomes [40]. Consistently, we have mutated miR-198 seed region and its efficient interaction with p62 protein is disrupted. Moreover, protein–protein interaction mechanisms could contribute to miR-198 recognition. Mutation of UBA, LIR, TB domains of p62 protein impaired its interaction with miR-198. However, the ZZ domain of p62 is shown to bind to small non-coding RNA [41], but in our study, p62^ZZ del^ protein still interacts with miR-198, therefore, we postulate that the small non-coding RNA interaction with p62 protein varies among different RNA species and is a highly selective process.

While our experiments explore autophagy, miRNA secretion, and vesicle uptake, we have not tested other non-coding RNAs and proteins that are secreted by autophagy so far. It will be informative to investigate whether p62 dependent autophagy also regulates other RNAs and proteins via vesicular secretion. It will also be relevant to explore possible molecular mechanisms that inherently induce p62 overexpression, and the exact EV groups for miRNA sequestration, which are destined to deliver to specific cell types.

Here, we provide evidences that autophagy specifies miRNA-protein complex into EVs. Previous work has focused mainly on interconnections between autophagy and cargo loading. For example, autophagy conducts the recruitment of miRNA processing enzyme DICER and the major miRNA effector AGO2 as miRNA-free entities for degradation [42]. In addition, miRNA itself, miR-224 was recruited to autophagosomes of HCC cells upon autophagy restoration [43]. Because autophagosomes can alternatively fuse with late endosomes, EV biogenesis and autophagy are proposed to be functionally connected [44]. However, until now, limited number of studies have elucidated the vesicle secretion of autophagosomes loaded cargos. In our study, we delineate a process for p62 protein directing miR-198 into autophagosomes and further secreted as EV enclosed miRNA. The regulation of miRNA function by a protein as seen for p62 could present a general principle of miRNA and protein control, complementing a well-recognized form of regulation such as post-translational modification and protein–protein interaction.

To note, miRNA expression profiles of HCC tissue are distinct from that of non-tumor tissues; most anti-proliferative miRNAs are found downregulated. Tumor suppressor miR-198 was previously detected in exosomes secreted from T-lymphocytes [40]. However, mechanisms regarding vesicle release amongst different cell types are de facto diversified and the vesicular miRNA secretion from hepatoma cells remains to be investigated. Here, we have demonstrated autophagy receptor protein, SQSTM1/p62 protein tasks as sponge carrier to absorb tumor suppressor miRNAs and shed off cell membrane as tumor derived EV. EVs from tumor cells are packaged with mRNAs and miRNAs [16] that markedly influence the tumor microenvironment or even enhance disease progression. We observed that miR-198-p62 was loaded in EVs, secreted from hepatoma cells and are transferrable to hepatic stellate cells (HSCs), the precursors of cancer-associated fibroblasts (CAFs). Using luciferase reporters as sensors for miR-198, we show that uptaken miR-198 is functional, inhibiting expression of the reporter which harbours the miR-198 target sequence. It is shown that the tumor surrounding CAF cells undergo increased p62 protein levels [45], indicating the possibility of p62 influx into CAF cells. As well, miR-198 is detected in serum samples of HCC patients [15]. These data all point to the involvement of not only paracrine uptake but also endocrine stimulation for vesicle uptake in vivo.

The future work will be needed to embed miRNA in EVs derived from hepatoma cells and study different cell types in the context of vesicle uptake in vivo, as tissues such as liver and spleen are composed of heterogeneous population of cells that could maintain different capability to accept vesicles. Therefore, the identification of potential recipient cell types of HCC EVs is the main research of interest to understand how liver cancer cells utilize self-secreted vesicles to grow and proliferate. Our study provides first evidences to the mechanism of miRNA-protein complex secretion and its uptake of accept cells, which could represent a general principle to supplement the well-recognized autophagy-mediated elimination of protein and tumor suppressor miRNAs. We predict that the secretory principle employed by p62 and miR-198 will be found to be more widespread in biology, especially among tumor cells.

## Supplementary Information

Below is the link to the electronic supplementary material.

Supplementary file1 (TIF 3573 KB) Figure S1 Vesicular of miR-198 release from hepatoma cells.Tet-on control, miR-21, miR-29a miR-145 and miR-198 expression system were established in HuH-7 cells. After dox treatment for 8 h, cells were lysed for Immunoblotting analysis using antibodies against LC3 and β-actin protein (A), and the cell supernatant were subject to vesicle isolation. The vesicular miR-21, miR-29a, miR-145 and miR-198 were analyzed by qPCR (B). Tet-on control and miR-198 expression system was established in Hep3B cells. After dox treatment for 8 h, both cellular (C) and vesicular (D) miR-198 levels were analyzed by qPCR. The supernatant from HuH-7 Tet-on miR-198 stable cells were collected, vesicles were isolated. Both vesicular and soluble miR-198 levels (E) were determined by qPCR. The miR-198 expression levels of Tet-on miR-198 stable cells were analyzed and compared to normal hepatocytes as shown parenchymal tissue (PC) (F)

Supplementary file2 (TIF 1818 KB) Figure S2 Autophagy affects miR-198 secretion into supernatant. Tet-On control and miR-198 cells were transfected with siATG5 and siATG7. After dox treatment for 24 h, the expression level of ATG5 (A) and ATG7 (B) were analyzed by qPCR. (C) EVs were isolated by the affinity column method and subjected to vesicle number calculation by NTA. The vesicle secretion was normalized by the confluency of cells in culture. (D) The vesicular miR-198 levels were analyzed by qPCR. (E) HuH-7 Tet-on and miR-198 stable cells were treated with dox for 24 h and BAF for 16 h. Cells were harvested for immunoblotting using antibodies against p62, β-actin and LC3 proteins

Supplementary file3 (TIF 7661 KB) Figure S3 p62 controls miR-198 sorting into EVs. HuH-7 cells were transfected with scRNA-Cy3, miR-29a-Cy3 or miR-198-Cy3. Fresh medium was changed twice at 6 h and 24 h post-transfection. Intracellular vesicles were stained using DiO dye. Cells were fixed by 4% PFA and viewed under confocal fluorescence microscope. The co-immunofluorescence imaging of intracellular vesicles and miRNAs were presented. The co-localization were calculated by Image J (A). HEK293 cells were stably transfected using miR-198 encoding plasmid initiated by CMV promoter. The stable cells were further overexpressed by p62 or LC3. miR-198 expression were analyzed by qPCR at 24 h post-transfection (B). Tet-On miR-198 expression system was established in HuH-7 p62KO cells. EVs were isolated by affinity column method using ExoEasy Maxi Kit (Qiagen, #76064) and subject to vesicle number calculation by NTA. The vesicle secretion (C) were normalized by the confluency of cells in culture. The vesicular miR-198 levels (D) were analyzed by qPCR. NS = no significance; ** means p<0.001

Supplementary file4 (TIF 1390 KB) Figure S4 miR-198 is selectively loaded by p62. HuH-7 Tet-On miR-198 stable cells were treated with dox for 8 h and cells were lysed for IP using antibody against p62 protein. Western blotting was performed to analyze p62 and LC3 protein in the IP precipitates (A). And p62 antibody based IP was performed in HuH-7 Tet-On miR-21 stable cells and HuH-7 Tet-On miR-29a stable cells. miR-21 and miR-29a levels in the IP precipitates were analyzed by qPCR (B). Furthermore, p62 or LC3 protein were overexpressed in the two stable cells lines by plasmid transfection. miR-21 (C) and miR-29a (D) expression were analyzed by qPCR.

Supplementary file5 (TIF 4877 KB) Figure S5 p62 mutation impairs its interaction with miR-198. Plasmids encoding different p62 truncated mutants were cotransfected with miR-198-Cy3 into HuH-7 cells. After 24 h, cells were fixed with methanol and immunostained using p62 antibodies. Cells were viewed under confocal fluorescence microscope and the co-immunofluorescence imaging of p62 UBA del and p62 PB1 del mutant (A) in miR-198-Cy3 enriched cells were presented. Blue, DNA; Red, scRNA; Green, LC3, p62 or intracellular vesicle as indicated. Scale bar = 10 µm. Co-localization (B) was analyzed by Image J as mentioned above.
