# Supplementary figures and images for: SQSTM1/p62 promotes miR-198 loading into extracellular vesicles and its autophagy-related secretion
Source: Hum Cell. 2022 Sep 1;35(6):1766–84. doi: 10.1007/s13577-022-00765-7 (PMC9515045; doi:10.1007/s13577-022-00765-7)

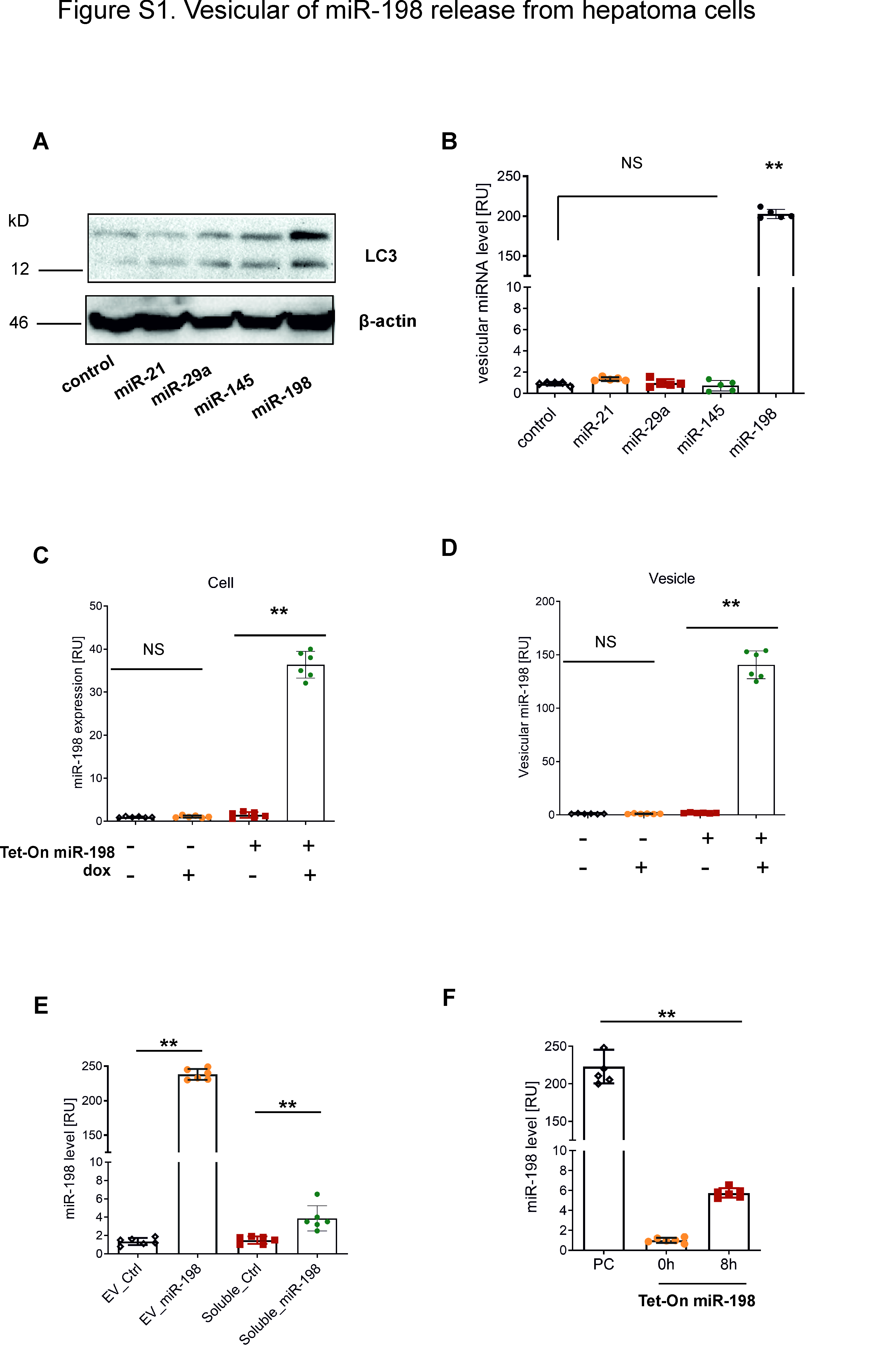

Supplement: Supplementary file 1 — Supplementary file1 (TIF 3573 KB) Figure S1 Vesicular of miR-198 release from hepatoma cells.Tet-on control, miR-21, miR-29a miR-145 and miR-198 expression system were established in HuH-7 cells. After dox treatment for 8 h, cells were lysed for Immunoblotting analysis using antibodies against LC3 and β-actin protein (A), and the cell supernatant were subject to vesicle isolation. The vesicular miR-21, miR-29a, miR-145 and miR-198 were analyzed by qPCR (B). Tet-on control and miR-198 expression system was established in Hep3B cells. After dox treatment for 8 h, both cellular (C) and vesicular (D) miR-198 levels were analyzed by qPCR. The supernatant from HuH-7 Tet-on miR-198 stable cells were collected, vesicles were isolated. Both vesicular and soluble miR-198 levels (E) were determined by qPCR. The miR-198 expression levels of Tet-on miR-198 stable cells were analyzed and compared to normal hepatocytes as shown parenchymal tissue (PC) (F) [file 13577_2022_765_MOESM1_ESM.tif]

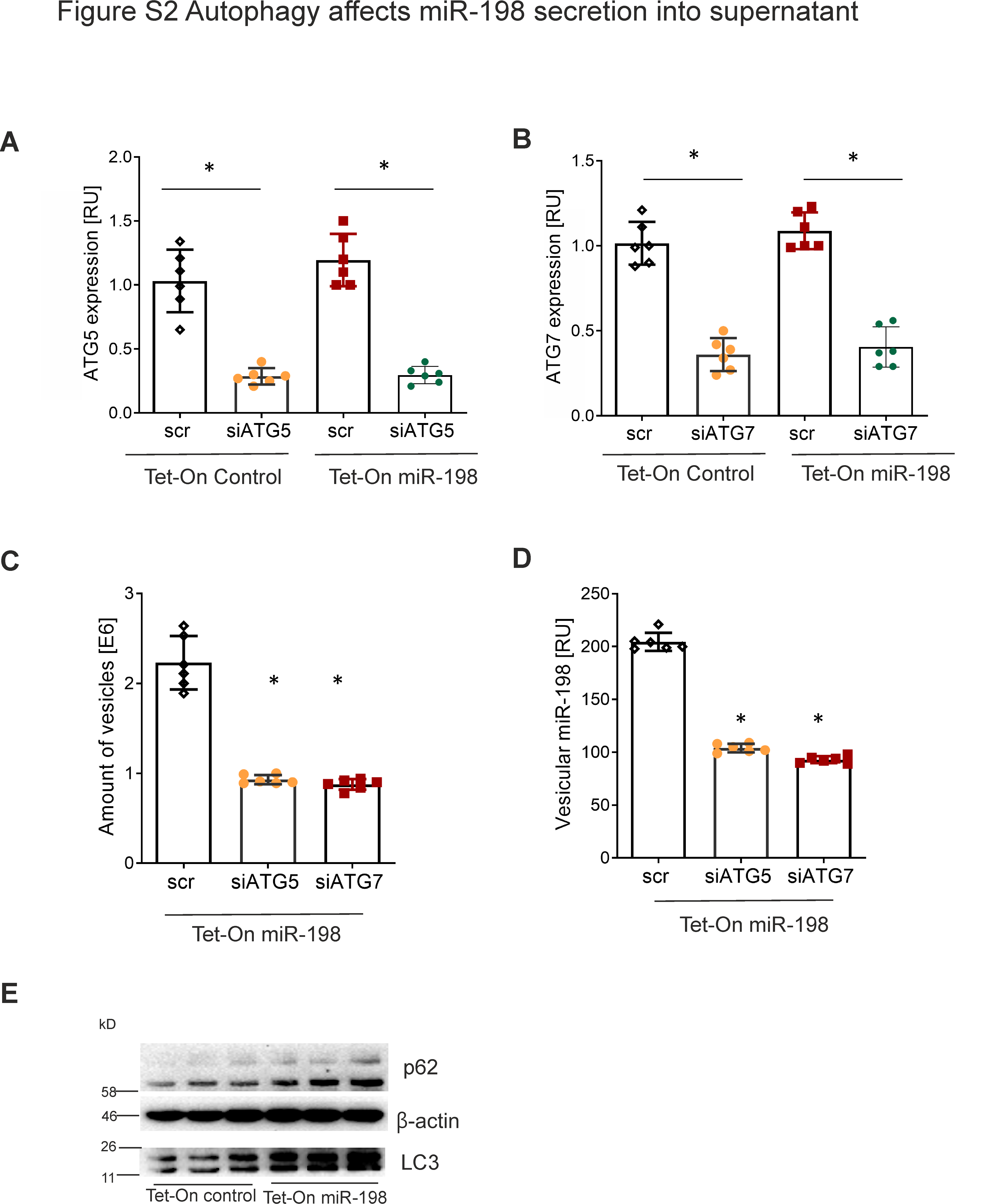

Supplement: Supplementary file 2 — Supplementary file2 (TIF 1818 KB) Figure S2 Autophagy affects miR-198 secretion into supernatant. Tet-On control and miR-198 cells were transfected with siATG5 and siATG7. After dox treatment for 24 h, the expression level of ATG5 (A) and ATG7 (B) were analyzed by qPCR. (C) EVs were isolated by the affinity column method and subjected to vesicle number calculation by NTA. The vesicle secretion was normalized by the confluency of cells in culture. (D) The vesicular miR-198 levels were analyzed by qPCR. (E) HuH-7 Tet-on and miR-198 stable cells were treated with dox for 24 h and BAF for 16 h. Cells were harvested for immunoblotting using antibodies against p62, β-actin and LC3 proteins [file 13577_2022_765_MOESM2_ESM.tif]

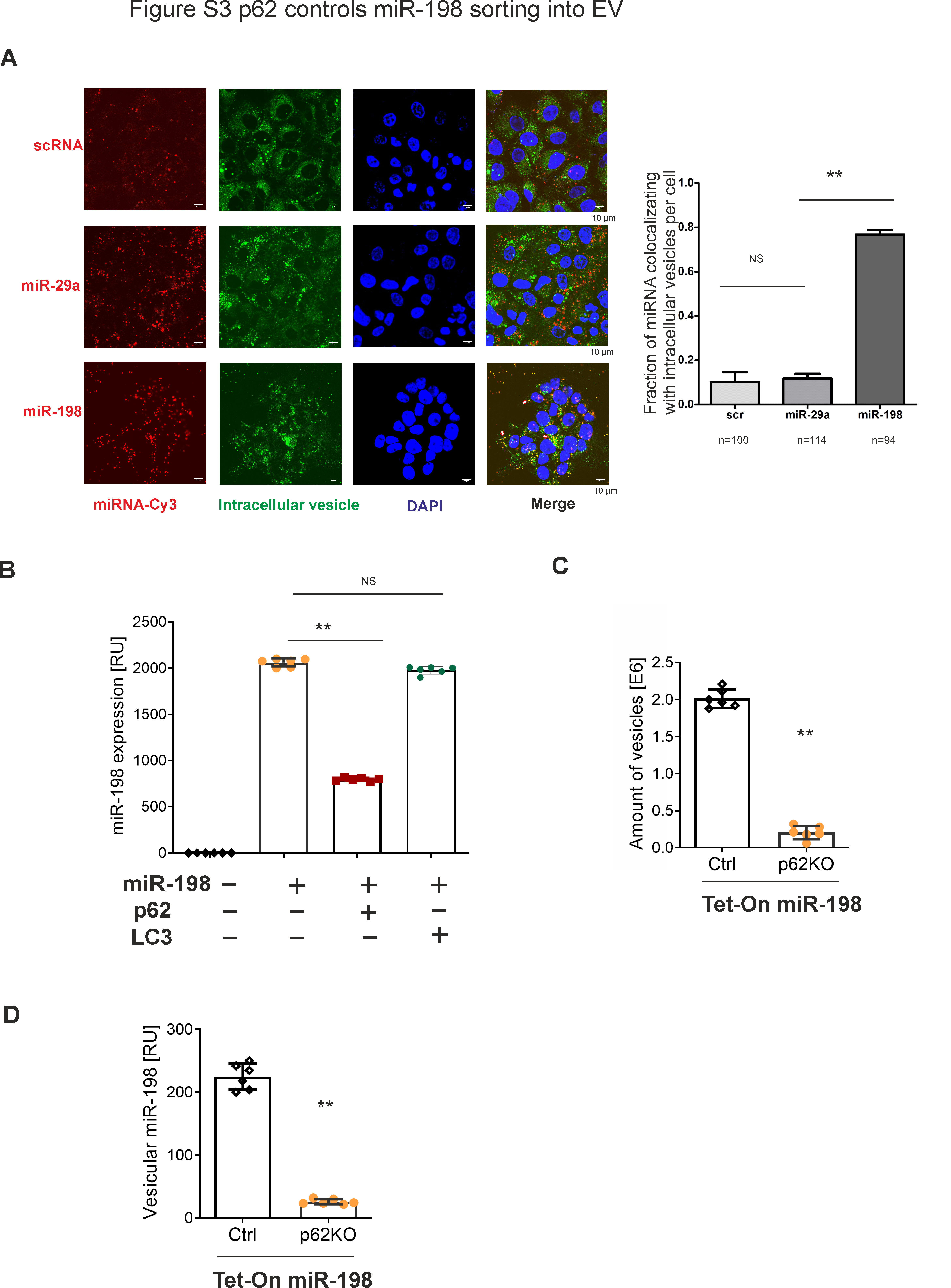

Supplement: Supplementary file 3 — Supplementary file3 (TIF 7661 KB) Figure S3 p62 controls miR-198 sorting into EVs. HuH-7 cells were transfected with scRNA-Cy3, miR-29a-Cy3 or miR-198-Cy3. Fresh medium was changed twice at 6 h and 24 h post-transfection. Intracellular vesicles were stained using DiO dye. Cells were fixed by 4% PFA and viewed under confocal fluorescence microscope. The co-immunofluorescence imaging of intracellular vesicles and miRNAs were presented. The co-localization were calculated by Image J (A). HEK293 cells were stably transfected using miR-198 encoding plasmid initiated by CMV promoter. The stable cells were further overexpressed by p62 or LC3. miR-198 expression were analyzed by qPCR at 24 h post-transfection (B). Tet-On miR-198 expression system was established in HuH-7 p62KO cells. EVs were isolated by affinity column method using ExoEasy Maxi Kit (Qiagen, #76064) and subject to vesicle number calculation by NTA. The vesicle secretion (C) were normalized by the confluency of cells in culture. The vesicular miR-198 levels (D) were analyzed by qPCR. NS = no significance; ** means p<0.001 [file 13577_2022_765_MOESM3_ESM.tif]

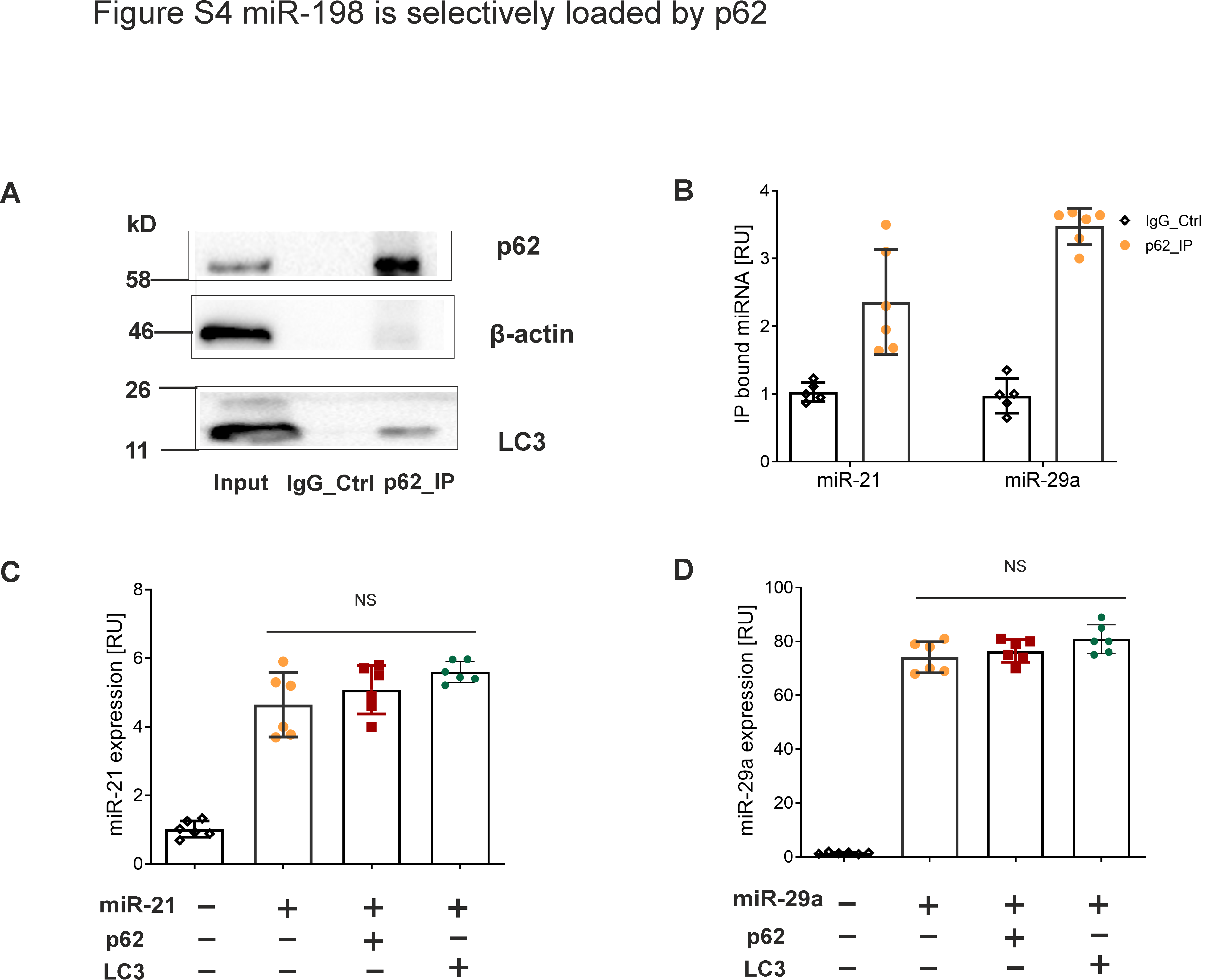

Supplement: Supplementary file 4 — Supplementary file4 (TIF 1390 KB) Figure S4 miR-198 is selectively loaded by p62. HuH-7 Tet-On miR-198 stable cells were treated with dox for 8 h and cells were lysed for IP using antibody against p62 protein. Western blotting was performed to analyze p62 and LC3 protein in the IP precipitates (A). And p62 antibody based IP was performed in HuH-7 Tet-On miR-21 stable cells and HuH-7 Tet-On miR-29a stable cells. miR-21 and miR-29a levels in the IP precipitates were analyzed by qPCR (B). Furthermore, p62 or LC3 protein were overexpressed in the two stable cells lines by plasmid transfection. miR-21 (C) and miR-29a (D) expression were analyzed by qPCR. [file 13577_2022_765_MOESM4_ESM.tif]

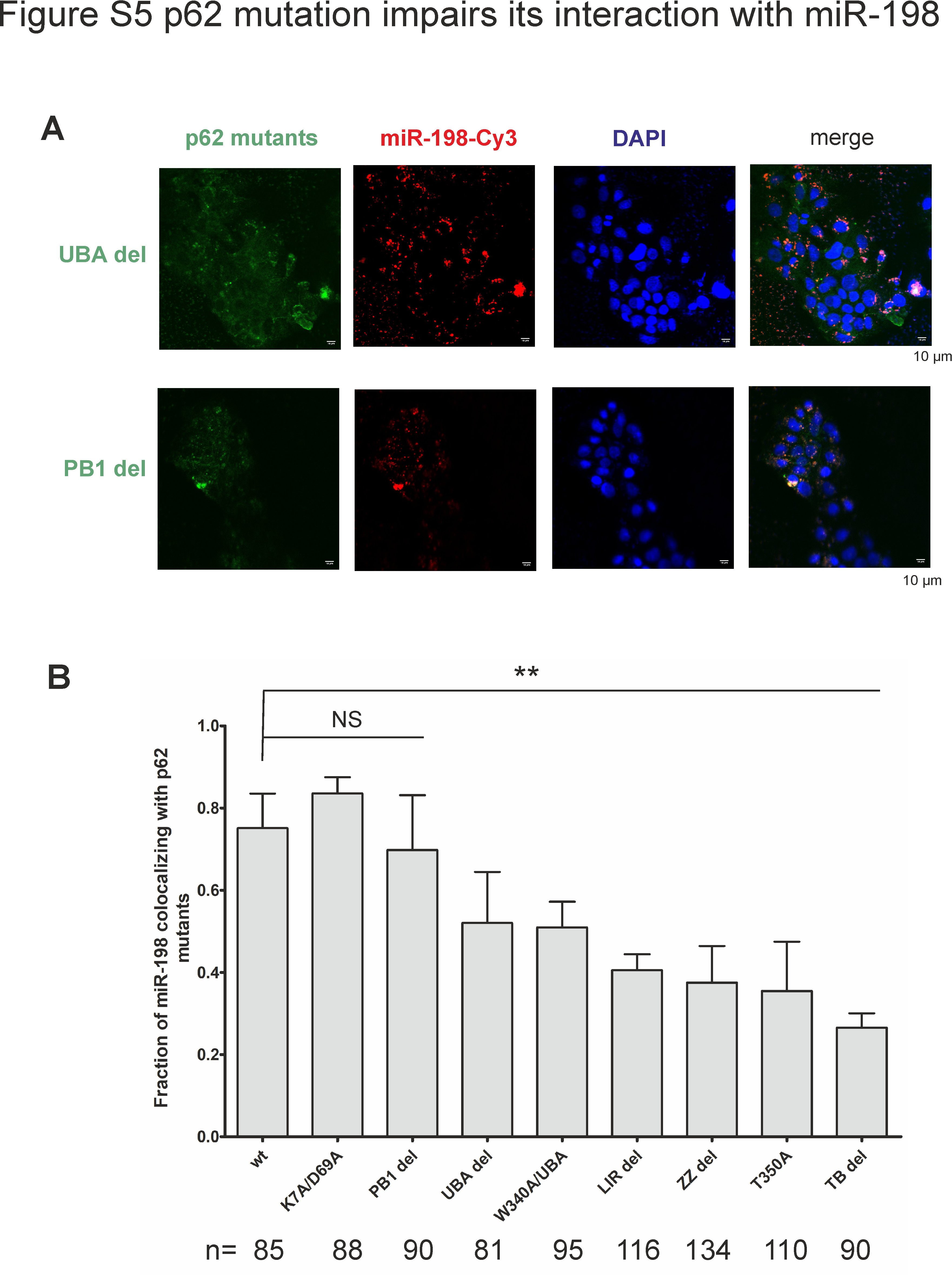

Supplement: Supplementary file 5 — Supplementary file5 (TIF 4877 KB) Figure S5 p62 mutation impairs its interaction with miR-198. Plasmids encoding different p62 truncated mutants were cotransfected with miR-198-Cy3 into HuH-7 cells. After 24 h, cells were fixed with methanol and immunostained using p62 antibodies. Cells were viewed under confocal fluorescence microscope and the co-immunofluorescence imaging of p62 UBA del and p62 PB1 del mutant (A) in miR-198-Cy3 enriched cells were presented. Blue, DNA; Red, scRNA; Green, LC3, p62 or intracellular vesicle as indicated. Scale bar = 10 µm. Co-localization (B) was analyzed by Image J as mentioned above. [file 13577_2022_765_MOESM5_ESM.tif]
